# Deep learning of movement behavior profiles and their association with markers of cardiometabolic health

**DOI:** 10.1186/s12911-024-02474-7

**Published:** 2024-03-13

**Authors:** Vahid Farrahi, Paul J Collings, Mourad Oussalah

**Affiliations:** 1 Institute for Sport and Sport Science, TU Dortmund University, Dortmund, Germany; 2 Physical Activity, Sport and Health Research Group, Department of Precision Health, Luxembourg Institute of Health, Strassen, Luxembourg; 3 Centre of Machine Vision and Signal Analysis, Faculty of Information Technology and Electrical Engineering, University of Oulu, Oulu, Finland

**Keywords:** Convolutional autoencoders, Cardiometabolic health, Clustering, Accelerometry, Wearables

## Abstract

**Background:**

Traditionally, existing studies assessing the health associations of accelerometer-measured movement behaviors have been performed with few averaged values, mainly representing the duration of physical activities and sedentary behaviors. Such averaged values cannot naturally capture the complex interplay between the duration, timing, and patterns of accumulation of movement behaviors, that altogether may be codependently related to health outcomes in adults. In this study, we introduce a novel approach to visually represent recorded movement behaviors as images using original accelerometer outputs. Subsequently, we utilize these images for cluster analysis employing deep convolutional autoencoders.

**Methods:**

Our method involves converting minute-by-minute accelerometer outputs (activity counts) into a 2D image format, capturing the entire spectrum of movement behaviors performed by each participant. By utilizing convolutional autoencoders, we enable the learning of these image-based representations. Subsequently, we apply the K-means algorithm to cluster these learned representations. We used data from 1812 adult (20–65 years) participants in the National Health and Nutrition Examination Survey (NHANES, 2003–2006 cycles) study who worn a hip-worn accelerometer for 7 seven consecutive days and provided valid accelerometer data.

**Results:**

Deep convolutional autoencoders were able to learn the image representation, encompassing the entire spectrum of movement behaviors. The images were encoded into 32 latent variables, and cluster analysis based on these learned representations for the movement behavior images resulted in the identification of four distinct movement behavior profiles characterized by varying levels, timing, and patterns of accumulation of movement behaviors. After adjusting for potential covariates, the movement behavior profile characterized as “Early-morning movers” and the profile characterized as “Highest activity” both had lower levels of insulin (*P* < 0.01 for both), triglycerides (*P* < 0.05 and *P* < 0.01, respectively), HOMA-IR (*P* < 0.01 for both), and plasma glucose (*P* < 0.05 and *P* < 0.1, respectively) compared to the “Lowest activity” profile. No significant differences were observed for the “Least sedentary movers” profile compared to the “Lowest activity” profile.

**Conclusions:**

Deep learning of movement behavior profiles revealed that, in addition to duration and patterns of movement behaviors, the timing of physical activity may also be crucial for gaining additional health benefits.

**Supplementary Information:**

The online version contains supplementary material available at 10.1186/s12911-024-02474-7.

## Introduction

In recent years, wearable technologies, such as accelerometer-based activity monitors, have facilitated the collection of extensive and detailed datasets on the full spectrum of human movement behaviors in free-living environments [1–3]. Due to their feasibility and applicability, many studies have utilized wearable activity monitors as an objective means of monitoring daily activities [4–7]. The robust evidence derived from these studies suggests human movement behaviors have considerable consequences on various health indicators in adults, including the risk of cardiometabolic diseases and mortality [7–9].

Adults engage in three types of movement behaviors in their daily living during waking hours—moderate-to-vigorous physical activity (MVPA), light intensity activities, and sedentary behaviors [9]. While the health benefits of MVPA are well-documented [10], the availability of device-based techniques has provided an unprecedented opportunity to explore whether and how the full spectrum of movement behaviors are related to health, especially when coupled with advanced machine learning and pattern recognition techniques [1, 4, 11]. Existing studies, utilizing wearable devices to monitor human movement behaviors, provide compelling evidence that excessive sedentary time, especially when combined with a low physical activity level, have detrimental effects on health [9]. Conversely, a higher level of physical activity from light intensity upwards is shown to be associated with improved cardiometabolic health [12]. Relying on such results, current public health guidelines recommend adults to accumulate a minimum of at least 150–300 min of moderate intensity physical activity each week, while minimizing total time spent in sedentary behaviors by incorporating any type of active behavior throughout the day to maximize health benefits [10].

The emergence of wearable activity monitors have facilitated the identification of previously unrecognized patterns of movement behaviors and their associations with health outcomes [2, 3, 13–15], leading to new insights and approaches for promoting a more active lifestyle. However, the study of relationships between human movement behaviors (measured with accelerometry or other ways) and health is a challenging task that necessitates using more innovative analytical approaches [1, 16]. Most existing studies to date have studied accelerometer data using classical statistical approaches such as regression analyses. However, machine learning approaches have the potential to better handle multidimensional accelerometer data [1, 4, 11, 15, 17, 18], potentially leading to identification of new insights and findings.

Recently, a number of analytical approaches have been proposed and used for studying the combined and joint associations of wearable device-estimated movement behaviors with different health indicators, rather than assessing them in isolation [4, 11, 12, 19]. Among these, statistical approaches like compositional data analysis and isotemporal substitution analysis have gained popularity in examining the interconnectedness of movement behaviors and their associations health outcomes [19–23]. By considering movement behaviors as compositional data, these approaches allow for a comprehensive understanding of their relative contributions to health markers and indicators, and how reallocations within these compositions may impact health outcomes [20, 23, 24]. More recently, machine learning and data-driven techniques have also been employed for movement behavior profiling and studying the joint associations of sedentary behaviors and physical activity with different health indicators [9, 25, 26]. For instance, machine-learned profiles of sedentary and activity behaviors, characterized by performing more physical activity at light-intensity upwards throughout an entire week, have been linked to better cardiometabolic health in adults [9]. Altogether, accumulating evidence arising from such studies suggests that not only the duration but also the timing and pattern of accumulation of sedentary behaviors and physical activity intensities could be linked to markers of cardiometabolic health [12, 27, 28].

However, there has been relatively little attention given to the health impacts of the timing and patterns of accumulation of movement behaviors, compared to the duration of movement behaviors [29]. This is partly because existing analytical approaches are not capable of processing accelerometer data in its original form due to its voluminous and dynamic nature, making it difficult to extract meaningful information using conventional statistical approaches. Most existing studies have been performed with averaged or aggregated values derived from accelerometer data [4, 11, 15, 27], while partly or completely ignoring both timing and patterns of accumulation of movement behaviors. Although some studies have incorporated variables and descriptors representing both levels and patterns of daily activities [4, 12, 15, 27, 28], new research based on novel analytical approaches is required to fully capture the complex dynamics of the duration, timing and patterns of accumulation of movement behaviors and assess their impacts on health.

Existing studies based on device-measured daily activities have consistently shown that any amount of physical activity could confer substantial cardiometabolic health benefits for adults [21]. Still, the patterns, variations, and timing of movement behaviors appear to be important and linked to a number of health markers in adults [4, 12, 27, 30, 31]. Although the findings of existing studies remain to be mixed, the timing and regularity of movement behavior (i.e., evening actives and morning actives) have also been found to be associated with cardiometabolic health markers [27, 30] and risk of mortality [30, 32, 33]. However, detailed guidelines about the timing and patterns of accumulation of sedentary and activity behaviors are still lacking [10], as it remains unclear whether timing and patterns of accumulation are as important as total durations of these behaviors in terms of health benefits [29].

In recent years, the field of machine learning has witnessed remarkable advances, with deep learning approaches excelling in various applications such as human activity recognition [34] and medical image analysis [35, 36]. Unlike traditional machine learning techniques [37–39], the most unique aspect of deep learning lies in its ability to automatically identify and learn representations from real-world data in their original form, without the need for hand-crafted feature engineering [36, 40]. These approaches can evaluate complex and high-dimensional data, enabling the identification of previously unrecognized patterns hidden in vast amounts of data [41]. This makes deep learning an excellent option for profiling accelerometer-measured daily movement behaviors without the need to reduce the accelerometer signal to averaged or aggregated values. Among the existing deep learning approaches, deep clustering based on convolutional autoencoders have recently gained interest due to their ability to learn data representations automatically with no or little supervision [42, 43]. Here, we generated image representations from the entire accelerometer outputs recorded during wear time, encompassing the duration, timing, and patterns of accumulation of movement behaviors. These movement behavior images were generated in such a way to encapsulate the entire waking movement behavior profile across seven measurement days from each participant into a single image. We then employed a novel deep learning clustering approach based on convolutional autoencoders to create profiles of accelerometer-estimated movement behaviors using these images, and examined whether and how these deep-learning-identified profiles of movement behaviors are associated with markers of cardiometabolic health.

## Methods

Data for this study were drawn from the National Health and Nutrition Examination Survey (NHANES). NHANES is a cross-sectional study that uses a complex, multistage probability design to obtain a representative sample of the USA civilian non-institutionalized population. For this study, data were drawn from the 2003/04 and 2005/06 NHANES cycles. The data collected in these cycles included completion of household interviews and surveys, an examination conducted in a mobile examination center, and wearing a hip worn accelerometer for the measurement waking activity behaviors. Further information about the NHANES study and recruitment process is detailed elsewhere [44].

### Study sample

All adult participants (20–65 years) who wore an accelerometer were considered eligible for inclusion in the present study. Participants with missing values for the biomarkers data or with insufficient valid accelerometer data were excluded. In total, there were 3688 adults eligible to wear an accelerometer in the total cycle sample of 10,348 participants of all ages. Of these, data from valid accelerometer and cardiometabolic outcomes for 1812 adults were available for the analyses.

### Accelerometer data collection and processing

All ambulatory participants attending the medical examination center were eligible for measurement of daily activities with a hip-worn accelerometer (Actigraph 7164; Actigraph, LLC, Fort Walton Beach, FLA). The Actigraph accelerometer is a small (5.1 × 4.1 × 1.5 cm), lightweight (0.4 kg) instrument that records integrated acceleration information as an activity count in counts per minute (cpm), providing an estimate of the intensity of bodily movement [45]. The activity counts are time and date stamped, so detailed data on the time, volume, and intensity of movement can be derived [45]. The accelerometer was worn on the right hip during waking hours (except for water-based activities) for 7 consecutive days.

#### Movement intensity categories

Accelerometry data were processed and cleaned using ‘rnhanesdata’ package in R [46]. Non-wear time intervals, defined as intervals of at least 60 consecutive minutes of 0 cpm with allowance for up to 2 min of observations of some limited movement (< 50 cpm) within these periods, were identified and flagged [45]. We conducted a visual inspection of all movement behavior images and identified that some wear time intervals lasting less than 120 min might have been non-wear periods, and incorrectly misclassified by the wear time detection method as wear time [45]. We therefore only included those wear time intervals that lasted more than 120 min to obtain better movement behavior images. This threshold was established through empirical analysis and visual inspection of the final movement behavior images. On average, the number of excluded bouts lasting less than 120 min among those who had accelerometry data was 3.1 per participant. The average duration of these bouts was 28.5 min. Participants with four or more valid days were considered eligible for inclusion in our study with each valid day was defined as ≥ 10 h of monitor wear time [45]. Accelerometer counts were used to mark all minute-by-minute activity counts using previously validated cut-points as either sedentary (< 100 cpm), light-intensity physical activity (100–1951 cpm), or MVPA (≥ 1952 cpm) [45, 47].

#### Duration of movement behaviors, uninterrupted sedentary bouts, and number of sedentary interruptions

Total duration of each activity category (min/day) was obtained by dividing time spent in each activity by the number of valid days. In addition to duration of movement behaviors, patterns of accumulation of sedentary time and number of sedentary interruptions are shown to be related to cardiometabolic health [12, 48]. A substantial body of literature indicates that excessive sedentary time is a health risk [49, 50]. Nevertheless, the exact threshold at which sedentary behavior becomes detrimental to health remains unclear. Yet, accumulating evidence from both randomized controlled trials and observational studies suggests that limiting sedentary time to 15–30 min may be beneficial for cardiometabolic health [12, 51]. Following consensus definition [52], all uninterrupted sedentary bouts lasting > 1 min were identified [52], and prolonged sedentary bouts were identified (15–30 min and ≥ 30-minutes sedentary bouts). Our rationale for selecting these two specific categories stems from accumulating evidence suggesting that sedentary time accumulated in bouts lasting more than 15–30 min may be considered prolonged and detrimental to cardiometabolic health [12, 51]. The percentage of sedentary time spent in each category (15–30 min and ≥ 30 min) was calculated by dividing the time spent in each sedentary bout category by the sum of the durations of all sedentary behaviors on valid days. Additionally, the number of transitions between sedentary bouts lasting > 1 min and active behaviors was identified, and divided by the sum of total time spend in sedentary behaviors on valid days to obtain the number of sedentary interruptions per sedentary hour. Sedentary break per hour is shown to be an appropriate metric specifically relevant to free-living behavior, reflecting the patterns of sedentary behaviors [53].

### Cardiometabolic markers

Participants’ height and weight were measured in the mobile examination center for the calculation of body mass index (BMI), and waist circumference was measured to the nearest 0.1 cm at the level of the iliac crest. Blood samples from the participants were analyzed for non-fasting high-density lipoprotein (HDL) cholesterol and C-reactive protein (CRP). The ratio of total to HDL cholesterol level (total/HDL cholesterol ratio) was derived as it provides a better prediction of cardiovascular disease risk than isolated lipid and lipoprotein levels [54]. One-half of the participants were sampled to attend the morning session [44]. Those participants attending the morning session were instructed to fast at least 9 h prior to their appointment time. Fasting blood samples were taken and analyzed for plasma glucose, insulin, low-density lipoprotein (LDL) cholesterol, and triglycerides. The homeostasis model assessment of insulin resistance (HOMA-IR) was calculated from fasting plasma glucose and insulin levels [55].

### Covariates

Participant self-reported their age, gender, marital status, ethnicity, and education level. Serum cotinine was measured to estimate the extent of active smoking and exposure to environmental tobacco smoke. The ratio of income to poverty was calculated based on family income values. Participant also completed two 24-hour diet-recall coupled with US Department of Agriculture food composition data to measure dietary intakes of total energy, saturated fat, and caffeine and alcohol consumption. Dichotomous variables were generated from self-reported medical history for diabetes, cardiovascular disease, and cancer.

### Deep learning of movement behavior for profile analysis

Profile analysis was conducted in three primary steps. Firstly, we used minute-by-minute activity counts to generate an image representing the full spectrum of movement behaviors for each participant who had valid accelerometry data. After creating one movement behavior image for each participant, profile analysis was performed using a Convolutional Autoencoder (CAE) and the K-means clustering algorithm. The CAE was trained on the movement behavior images to learn the image representation (i.e., latent variables), which were then fed to the K-means clustering to group the participants in such a way that participants in the same group exhibited the most similar movement behaviors, while demonstrating the most dissimilarity in terms of movement behaviors from the participants in other groups. These three steps are explained in more detail below.

#### Representation of movement behaviors as images

**Fig. 1:**
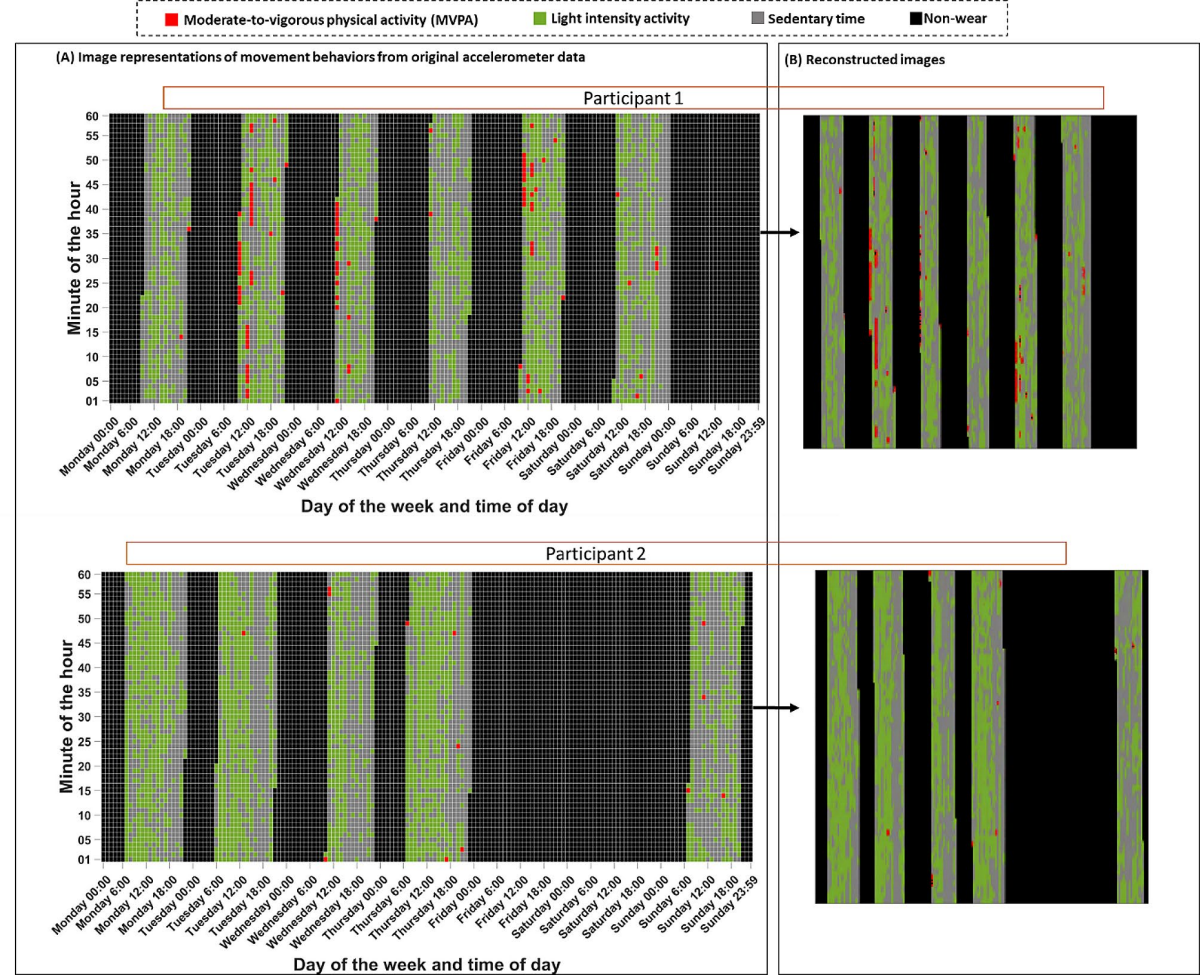
Examples of movement behavior images used as input for convolutional autoencoders. Panel (**A**) displays the movement behavior profile images created from accelerometer activity counts per minute during valid measurement periods over the course of 7 measurement. Panel (**B**) displays the reconstructed movement behavior profile images from the learned latent variable using convolutional autoencoders. Participants with four or more valid days were considered eligible for inclusion in our study with each valid day was defined as ≥ 10 h of monitor wear time. Accelerometer outputs (counts per minute [cpm]) were classified using previously validated cut-points as either sedentary (< 100 cpm), light-intensity physical activity (100–1951 cpm), or moderate-to-vigorous physical activity (MVPA, ≥ 1952 cpm). Note that all the axes’ labels and grid lines were removed from the images when creating movement behavior images for training the convolutional autoencoders. One movement behavior image was created for each participant

Considering that the accelerometer outputs activity counts each minute, up to 10,080 (7 day × 24 h × 60 min = 10,080) date- and time-stamped data points, categorized as MVPA, light intensity activity, or sedentary time were available for each participant. We sorted these data points by day of the week, from Monday to Sunday, and represented them as a 2D matrix with 168 rows (7 days × 24 h) and 60 columns (minutes per hour). Recorded MVPA, light intensity activity, and sedentary minutes were color-coded and non-wear time intervals were represented with in black. Figure 1 depicts two examples of movement behavior images created based on minute-by-minute accelerometer data.

#### Convolutional autoencoders

We utilized CAE for deep learning of movement behavior profiles. CAEs are a type of deep neural network with an encoder and a decoder layer [42, 56, 57]. The encoder converts input data into a compressed representation, called latent variables, while the decoder reconstructs the original input data from these latent variables. The network is trained to compress the data into a low-dimensional vector at the bottleneck and reconstruct the input data. Deep clustering with convolutional autoencoders leverages convolutional layers to effectively generate low-dimensional representations of high-dimensional data [42, 56]. This approach allows for unsupervised learning of data representations. The compressed representations are then used for clustering algorithms, such as K-means clustering [56].

**Fig. 2:**
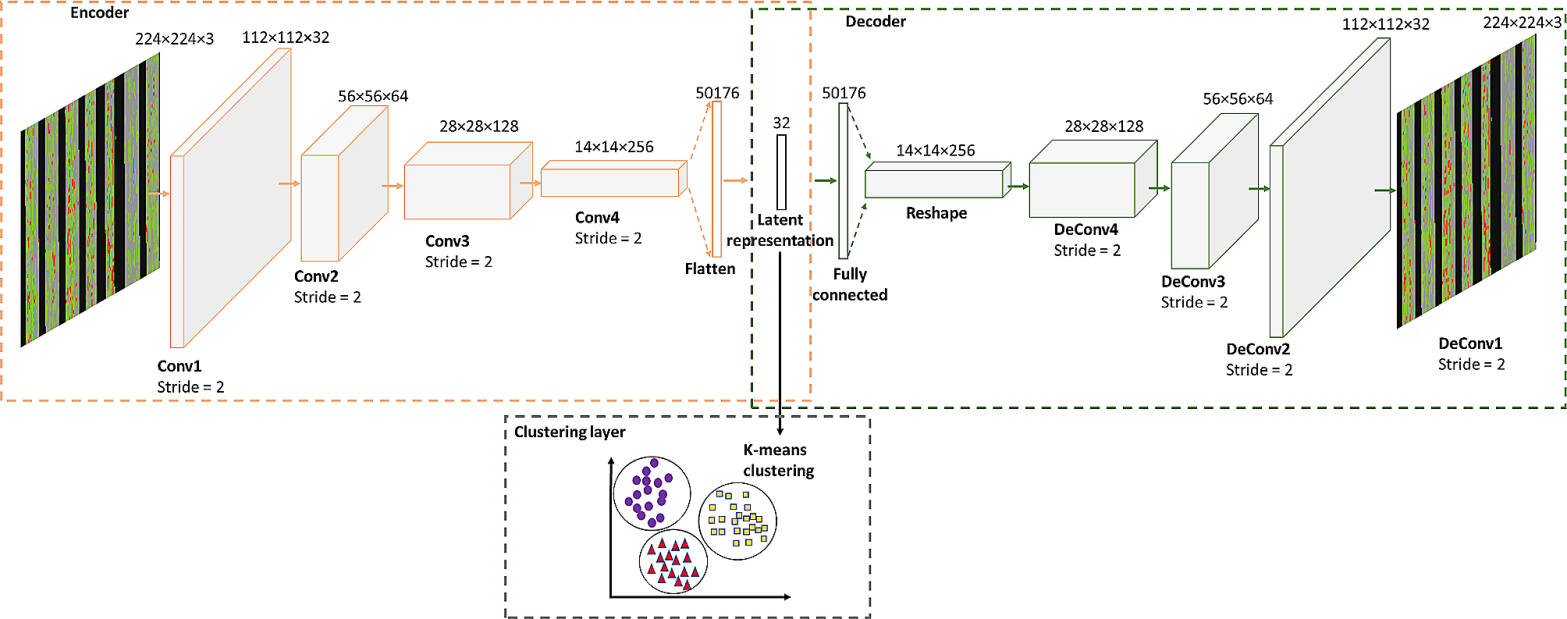
The structure of convolutional autoencoders employed for deep clustering of movement behavior images. The encoder network converts the input data into a compressed representation, and the decoder network reconstructs the original input data from the learned compressed representation. The encoder network comprises convolutional layers, and the decoder network comprises deconvolutional layers (or convolutional transpose layers). In the middle lies a fully connected autoencoder, whose embedded layer consists of 32 neurons, creating the latent representation. The network was trained in an end-to-end manner. The clustering layer received the latent representations as input and employed K-means clustering to divide the data into non-overlapping clusters

Figure 2 shows the architecture of the CAE used for deep learning of movement behaviors profiles from the generated images. This network represents a variation of the CAE introduced by Gue et al. [56], that is widely utilized in the existing literature due its efficiency in feature learning. In this architecture, only convolutional layers are layered on the input images to extract hierarchical features [56]. There is a fully connected autoencoder with an embedded layer composed of user-specified neurons. The rest are convolutional layers and convolutional transpose layers (some work refers to as Deconvolutional layer). In our study, the CAE comprised four convolutional layers and four deconvolutional layers, which encoded the images into 32 latent variables.

To train the CAE network, all the generated movement behavior images were normalized and resized to 224 × 224 pixels, and then were fed into the CAE. We empirically determined the optimal input size by progressively training the network, starting from 8 × 8 pixels and doubling the input dimensions up to 2048 × 2048 pixels. In each repetition, we systematically tested encoding images into various numbers of latent variables, ranging from 8 to 2048 latent variables, with each iteration doubling the size of the latent variable. These empirical tests indicated that the input size of 224 × 224 pixels with 32 latent variables sufficed for the CAE to learn the image representation appropriately, as shown in Fig. 1 (panel (B)), preserving the temporal distribution of sedentary and physical activity bouts. We used an Adam optimizer with a mini-batch size of 32 and fixed the number of epochs to 200.

#### K-means clustering with the learned latent variables

Clustering analysis was performed with the K-means clustering algorithm. K-means partitions the data into a user-defined number (K) of disjoint clusters based on the input variables (features) [58]. The cost function is optimized such that objects within the same cluster have maximized similarity to each other and minimized similarity to objects assigned to other clusters [58]. We included the learned 32 latent variables from the CAE as input to the K-means clustering analysis, using the K-means + + strategy for centroid initialization [59]. Unlike random selection of centroids, K-means + + selects the initial cluster centers that are as far apart as possible [59]. This approach reduces the risk of converging to a local minimum and enhances the algorithm’s ability to discover meaningful clusters in the data [59]. To determine the optimal number of clusters for our analysis, we employed the “elbow method” [60] and silhouette analysis [61], which are two commonly-used approaches for cluster quality analysis. The elbow method involves selecting the optimal number of clusters based on a trade-off between a reasonable number of clusters and the minimization of within-cluster differences [60]. Meanwhile, silhouette analysis allows us to examine the separation distance between the resulting clusters [61]. This measure spans from − 1 to 1, with values closer to 1 indicating that the sample is more distant from the neighboring clusters. We visualized silhouette scores for all data points to assess the appropriateness of the number of clusters.

### Statistical analysis

#### Characteristics of movement behavior profiles

Descriptive statistics were calculated for the sample population as well as for each movement behavior profile. After identifying the movement behavior profiles, significance of difference in mean time spent in MVPA, light intensity activities, and sedentary time among the identified profiles were examined with one-way analysis of covariance (ANCOVA) with adjustment for the effects of age, gender, ethnicity, marital status, and income to poverty ratio. Comparisons were also made for the percentage of time spent in 15–30 min and ≥ 30-minute sedentary bouts, and the number of sedentary interruptions per hour. When the differences between profiles were found to be statistically significant (*p* < 0.05) in ANCOVA tests, pairwise comparison was performed with Tukey post-hoc tests.

To capture the temporal distribution of movement behaviors, we calculated and visually represented the percentage of participants within each profile who spent their time engaged in MVPA, light intensity activities, and sedentary time for each minute from Monday to Sunday. This approach allowed us to effectively demonstrate the temporal distribution of movement behaviors in each identified profile and compare the differences among the profiles.

#### Associations with markers of cardiometabolic health

Multiple linear regression models were used to assess the association between profile membership with each of the cardiometabolic health markers. All non-normally distributed cardiometabolic markers were log-transformed prior to inclusion in the regression analyses to meet the assumption of normal distribution. We defined the profile with lowest activity levels (both MVPA and light intensity activities) as the referent profile, and then compared the differences in cardiometabolic health markers among the profiles to the reference profile. The regression models for each cardiometabolic marker were adjusted for significant confounders identified through outcome-specific backward elimination(retained at *P* < 0.2 [62]).

## Results

### Participants

A total of 1812 NHANES participants aged 20–65 years provided valid acceleration data, along with all the cardiometabolic markers required for the present study. Descriptive statistics for the participants included in the analysis, both overall and by the four waking activity behavior profiles identified, are presented in Table 1. The mean age of participants was 43.1 (14.3) years, and 53.5% were female. The average daily wear time (SD) of the accelerometer was 14.2 (1.5) hours per day.

**Table 1:** Characteristics of the study population overall, and by the four identified movement behavior profiles

Variable	Full sample (*N* = 1812)	Lowest activity (*N* = 351)	Early-morning movers (*N* = 595)	Least sedentary movers (*N* = 552)	Highest activity (*N* = 314)
*Demographics*					
Age, years	43.1(12.5)	40.4 (13.0)	44.8 (12.3)	41.4 (12.7)	45.8 (11.0)
Sex					
Male	842 (46.5%)	154 (43.9%)	265 (44.5%)	229 (41.5%)	194 (61.8%)
Female	970 (53.5%)	197 (56.1%)	330 (55.5%)	323 (58.5%)	120 (38.2%)
Race/Ethnicity					
Non-Hispanic white	907 (50.1%)	152 (43.3%)	344 (57.8%)	249 (45.1%)	162 (51.6%)
Non-Hispanic black	332 (18.3%)	79 (22.5%)	102 (17.1%)	94 (17.0%)	57 (18.2%)
Mexican American	423 (22.3%)	76 (21.7%)	114 (19.2%)	158 (28.6%)	75 (23.9%)
Other	150 (8.3%)	44 (12.5%)	35 (5.9%)	51 (9.2%)	20 (6.4%)
Education					
Less than 12 years	396 (21.9%)	86 (24.5%)	104 (17.5%)	140 (25.4%)	66 (21.0%)
12 years	427 (23.6%)	88 (25.1%)	112 (18.8%)	136 (24.6%)	91 (29.0%)
Over high school	989 (54.6%)	177 (50.4%)	379 (63.7%)	276 (50.0%)	157 (50.0%)
Marital status					
Married/cohabiting	1271 (70.2%)	202 (57.7%)	439 (73.8%)	385 (69.7%)	245 (78.0%)
Divorced/Widowed	284 (15.7%)	65 (18.6%)	86 (14.5%)	86 (15.6%)	47 (15.0%)
Never married	256 (14.1%)	83 (23.7%)	70 (11.8%)	81 (14.7%)	22 (7.0%)
Income to poverty ratio	3.0 (1.6)	2.6 (1.6)	3.2 (1.6)	2.8 (1.6)	3.2 (1.5)
*Lifestyle factors, dietary, and diseases*					
Smoking status (serum cotinine)					
Non-smoker, < 10 ng/dL	1372 (75.7)	240 (68.4)	483 (81.2)	415 (75.2)	234 (74.5)
Light smoker, 10-<100 ng/dL	108 (6.0)	25 (7.1)	24 (4.0)	37 (6.7)	22 (7.0)
Moderate smoker, 100-<300 ng/dL	213 (11.8)	59 (16.8)	56 (9.4)	62 (11.2)	36 (11.5)
Heavy smoker, ≥ 300 ng/dL	119 (6.6)	27 (7.7)	32 (5.4)	38 (6.9)	22 (7.0)
Alcohol consumption, grams/day	8.6 (21.9)	7.2 (18.9)	9.0 (24.1)	8.9 (22.3)	9.0 (19.8)
Dietary energy intake, kcal	2198.3 (797.2)	2215.2 (836.7)	2215.4 (777.0)	2088.5 (758.7)	2336.3 (833.8)
Total saturated fat, gram	27.4 (13.1)	27.6 (14.0)	27.6 (12.8)	25.9 (12.4)	29.3 (13.7)
Total caffeine intake, milligram	163.3 (192.9)	154.6 (208.6)	161.9 (180.4)	154.6 (186.2)	190.1 (207.5)
Diseases					
Cardiovascular diseases	94 (5.2%)	23 (6.6%)	26 (4.4%)	28 (5.1%)	17 (5.4%)
Diabetes	138 (7.6%)	34 (9.7%)	33 (5.5%)	49 (8.9%)	22 (7.0%)
Ever had cancer or malignancy	89 (4.9%)	18 (5.1%)	24 (4.0%)	25 (4.5%)	22 (7.0%)
*Cardiometabolic markers*					
Insulin, pmol/L	47.8 (29.5, 81.1)	54.2 (34.4, 90.8)	42.5 (25.9, 72.5)	49.9 (31.6, 83.0)	47.5 (29.4, 77.1)
Triglycerides, mmol/L	1.3 (0.9, 1.9)	1.3 (0.9, 2.0)	1.2 (0.9, 1.8)	1.3 (0.9, 2.0)	1.2 (0.8, 1.8)
HOMA-IR	11.5 (6.7, 21.0)	13.5 (8.0, 23.5)	10.2 (5.8, 18.7)	11.8 (7.2, 21.4)	11.7 (7.1, 20.5)
Plasma glucose, mmol/L	5.3 (4.9, 5.8)	5.4 (5.0, 5.8)	5.3 (4.9, 5.7)	5.3 (4.9, 5.7)	5.4 (5.1, 5.8)
CRP, mg/dL	0.2 (0.1, 0.5)	0.2 (0.1, 0.6)	0.2 (0.1, 0.6)	0.2 (0.1, 0.5)	0.2 (0.1, 0.4)
Total/HDL cholesterol ratio	3.6 (2.9, 4.5)	3.7 (2.9, 4.5)	3.6 (2.9, 4.6)	3.6 (3.0, 4.5)	3.7 (2.9, 4.6)
LDL, mmol/L	3.0 (0.9)	3.0 (1.0)	3.1 (0.9)	3.1 (0.9)	3.1 (0.9)
Waist circumference, cm	96.2 (86.7, 107.0)	96.4 (87.3, 108.2)	95.6 (86.2, 105.0)	96.3 (87.0, 107.2)	97.8 (87.0, 108.7)
BMI, kg/m^2^	27.8 (24.2, 32.0)	28.0 (24.1, 32.9)	27.4 (24.1, 31.3)	28.0 (24.4, 32.1)	27.8 (24.4, 31.9)

Values are mean (SD) or count (%), and median (interquartile range) for the cardiometabolic markers. BMI = body mass index, CRP = C-reactive protein, LDL = low-density lipoprotein, HDL = high-density lipoprotein, HOMA-IR = Homeostasis insulin resistance (HOMA-IR)

### Deep clustering of waking activity behaviors and the optimal number of clusters

All movement behavior profile images were used to train the CAE network, and the learning curve for the CAE encoding the images into 32 latent variables is shown in Supplementary material, Figure S1. The cluster analysis was performed with the learnt latent variables. The within-cluster sums for K-means cluster analysis with the 32 learned latent variables from CAE and the number of clusters ranging from 1 to 50 are shown in Supplementary material, Figure S2.

According to both the “elbow method”, three or four clusters seemed to be appropriate (see Supplementary material, Figure S2). Similarly, the visualization of silhouette scores within each cluster suggested that three or four clusters were appropriate, as they demonstrated a more balanced size and acceptable separation (see Supplementary material, Figures S3–S11). To determine the optimal number of clusters, we repeated the clustering process with both three and four clusters. During each repetition, we analyzed the number of participants assigned to each cluster. We also assessed which clustering solution resulted in more apparent differences in the average time spent in MVPA, light intensity activities, and sedentary time. Additionally, we also examined the differences in the percentage of time spent in 15–30 min and ≥ 30-minute sedentary bouts, along with the number of sedentary interruptions per hour. We selected four as the optimal number of clusters (or profiles) because of the balanced distribution of participants across clusters and to maximize the differences in between them.

#### Comparison of movement behavior profiles/clusters

**Fig. 3:**
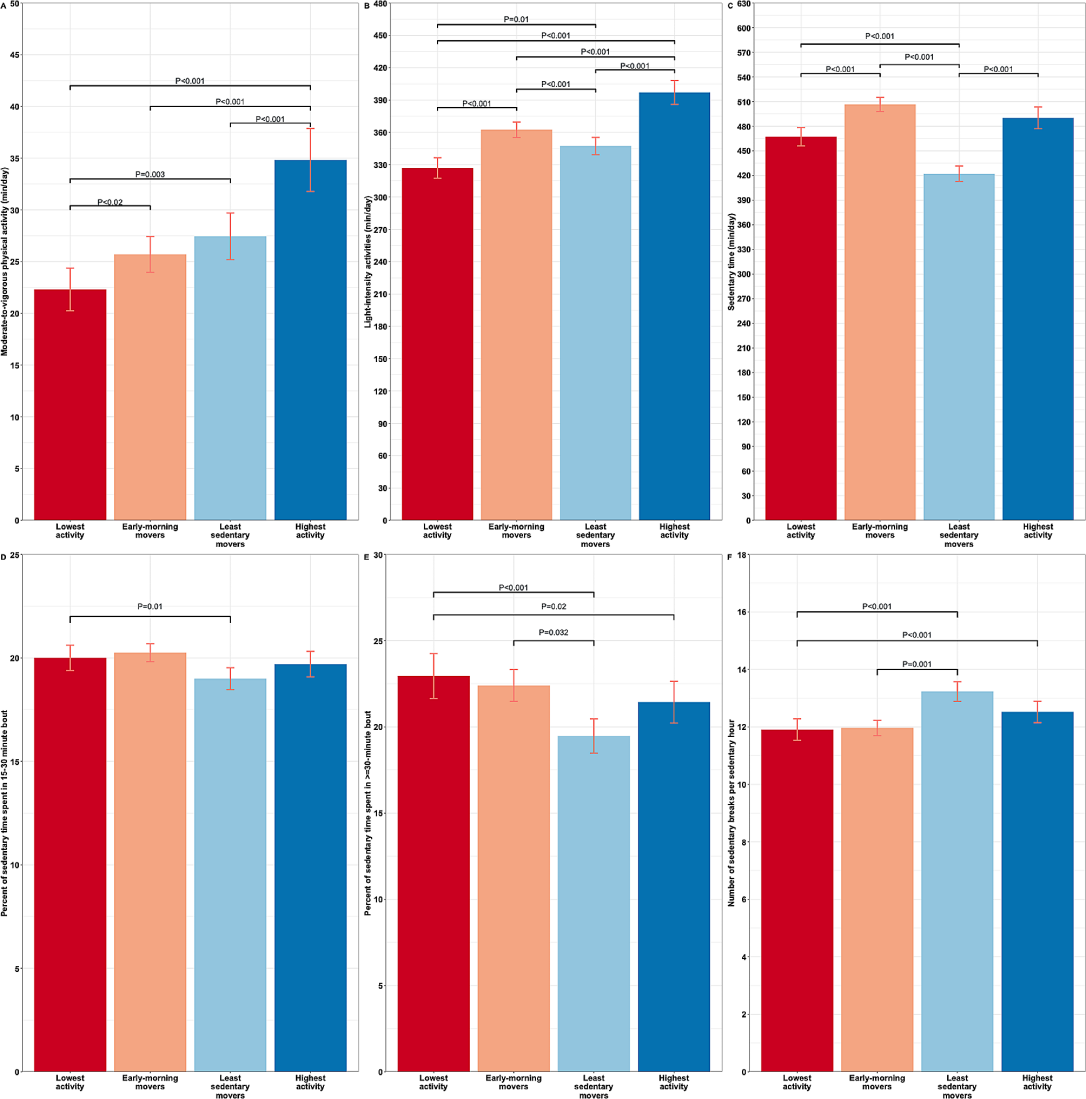
Total duration of (**A**) moderate-to-vigorous physical activity (MVPA), (**B**) light intensity activities, and (**C**) sedentary time, and percent of time spent in 15–30 min (**D**) and ≥30-minute (**E**) sedentary bout length, and number of sedentary breaks per sedentary hour (**F**) in the four identified movement behavior profiles. The bars represent the mean, and error bars indicate 95% confidence intervals. Differences were examined with analysis of covariance (ANCOVA) with adjustment for the effects of age, gender, ethnicity, marital status, and income to poverty ratio, and only significant pairwise comparison with p-values < 0.05 are shown on the graphs

The durations of MVPA, light intensity activities, and sedentary time, along with the percentage of sedentary time spent in different bout length and the number of sedentary interruptions for these clusters are presented in Fig. 3. The identified profiles exhibited varying and statistically different levels of MVPA, light intensity activities, and sedentary time. Differences were also observed in the percentage of sedentary behaviors spent in different bout length and the number of sedentary interruptions between the identified profiles.

**Fig. 4:**
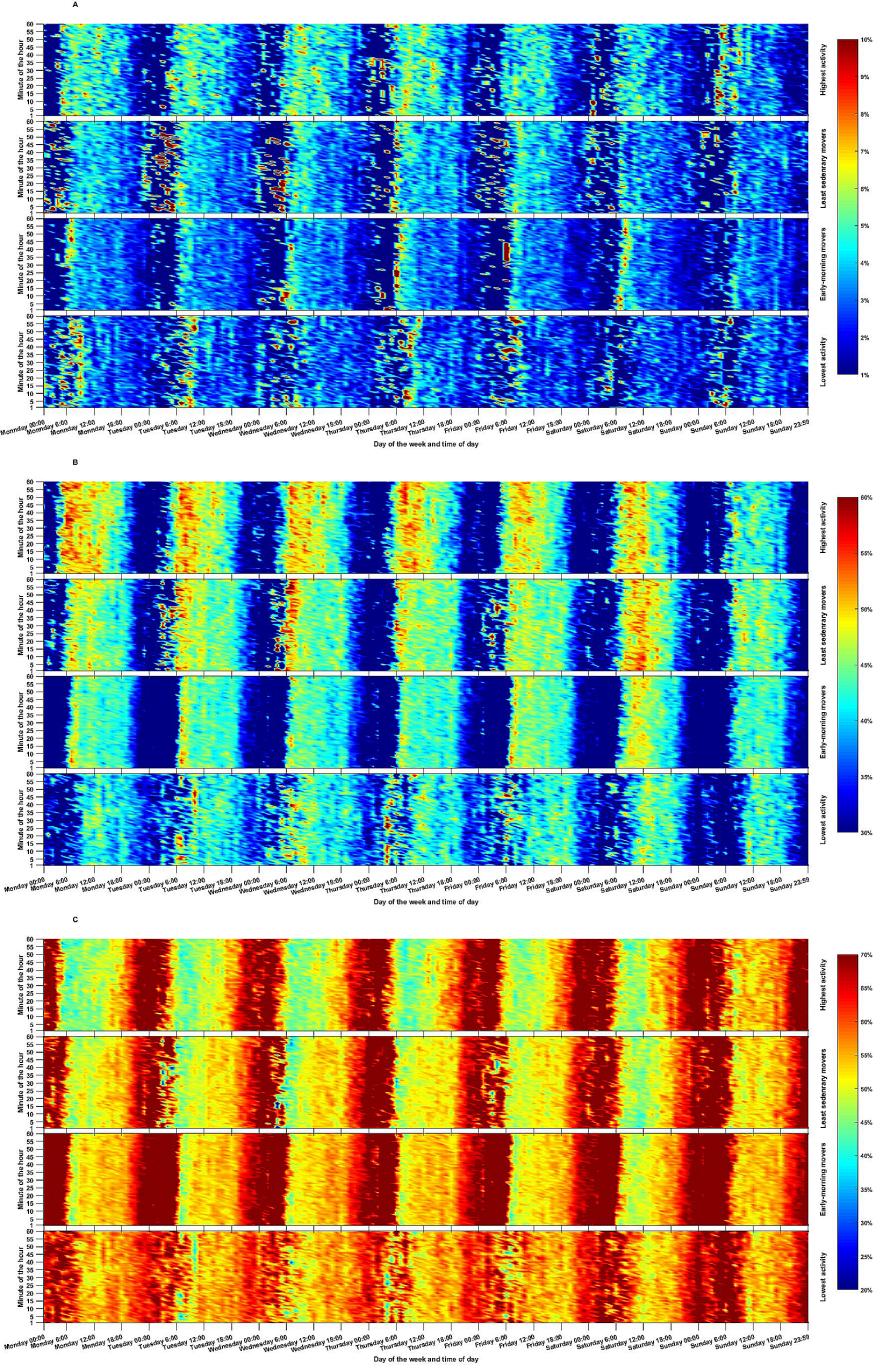
Temporal distribution of (**A**) moderate-to-vigorous physical activity (MVPA), (**B**) light intensity activities, and (**C**) sedentary time among the four identified profiles. The heatmaps illustrate the percentage of participants within each profile engaged in MVPA, light intensity activities, and sedentariness for each minute from Monday to Sunday. Warmer colors indicate a higher percentage of participants engaging in the specific movement behavior during those time intervals, while colder colors indicate lower participation rates. The values are interpolated for better interpretation

Figure 4 displays the temporal distribution of MVPA, light intensity activities, and sedentary time, represented by minute-by-minute percentage distributions. Overall, all movement behavior profiles tended spend more time sedentary during the evening hours after ~ 17 pm, and less time in MVPA and light-intensity activities.

#### Cluster 1: “Lowest activity” (*N* = 351)

This profile showed the lowest level of MVPA and light-intensity activities among all the profiles (*P* < 0.02 for all pairwise comparisons). Additionally, they spent a higher amount of time engaged in sedentary bouts lasting 30 min or more (*P* < 0.05 for all pairwise comparisons). In comparison to all other movement behavior profiles, the temporal distribution of MVPA, light intensity activities, and sedentary time appeared to be relatively more varied.

#### Cluster 2: “Early-morning movers” (*N* = 595)

This cluster, on average, had slightly higher duration of MVPA (*P* < 0.02), light intensity activities (*P* < 0.001), and sedentary time (*P* < 0.001) than “Lowest activity”. However, there were no statistically significant differences in the number of sedentary interruptions and the percentage of time spent in sedentary bouts lasting 15–30 min and ≥ 30-minute sedentary bouts. In terms of timing of movement behaviors, “Early-morning movers” consistently displayed a tendency to engage in MVPA and light-intensity activities during the early morning hours, specifically between approximately 6–8 am, from Monday to Friday. On Saturdays, their engagement in MVPA and light intensity activities was higher between approximately 7–12 am.

#### Cluster 3: “Least sedentary movers” (*N* = 552)

This profile had, on average, the least time spent in sedentary behavior compared to other identified profiles (*P* < 0.05). In comparison to the “Lowest activity” and “Early-morning movers” profiles, the “Least sedentary movers” profile had a higher number of sedentary interruptions (*P* < 0.001 and *P* = 0.001, respectively) and spent relatively less time in sedentary bouts lasting ≥ 30 min (*P* < 0.001 and *P* = 0.032, respectively). Regarding the timing of movement behaviors, the “Least sedentary movers” consistently displayed a tendency to be relatively more sedentary during the morning hours (6–12 am) compared to the afternoon hours (12–18 pm). Conversely, they engaged in more MVPA and light-intensity activities in the morning (6–12 am) than in the afternoon (12–18 pm).

#### Cluster 4: “Highest activity” (*N* = 314)

This profile exhibited the highest level of MVPA and light intensity activities compared to all other profiles (*P* < 0.001 for all pairwise comparisons). Additionally, they had a comparable level of sedentary behaviors when compared to the “Lowest activity” and “Early-morning movers” profiles but higher (*P* < 0.001) than the “Least sedentary movers” profile. The “Highest activity” profile did not show any statistically significant differences in the percentage of time spent in sedentary bouts lasting 15–30 min and ≥ 30 min sedentary bouts, as well as the number of sedentary interruptions, in comparison to the “Least sedentary movers” profile. However, when compared to the “Lowest activity” and “Early-morning movers” profiles, they spent relatively less time in sedentary bouts lasting ≥ 30 min (*P* = 0.02). Regarding the timing of movement behaviors, the “Highest activity” performed their MVPA and light intensity activities mostly in daytime between ~6 am-16 pm. They were also relatively less sedentary during those hours. The only difference was on Sunday when the “Highest activity” profile tended to remain mostly sedentary.

### Association analysis

**Fig. 5:**
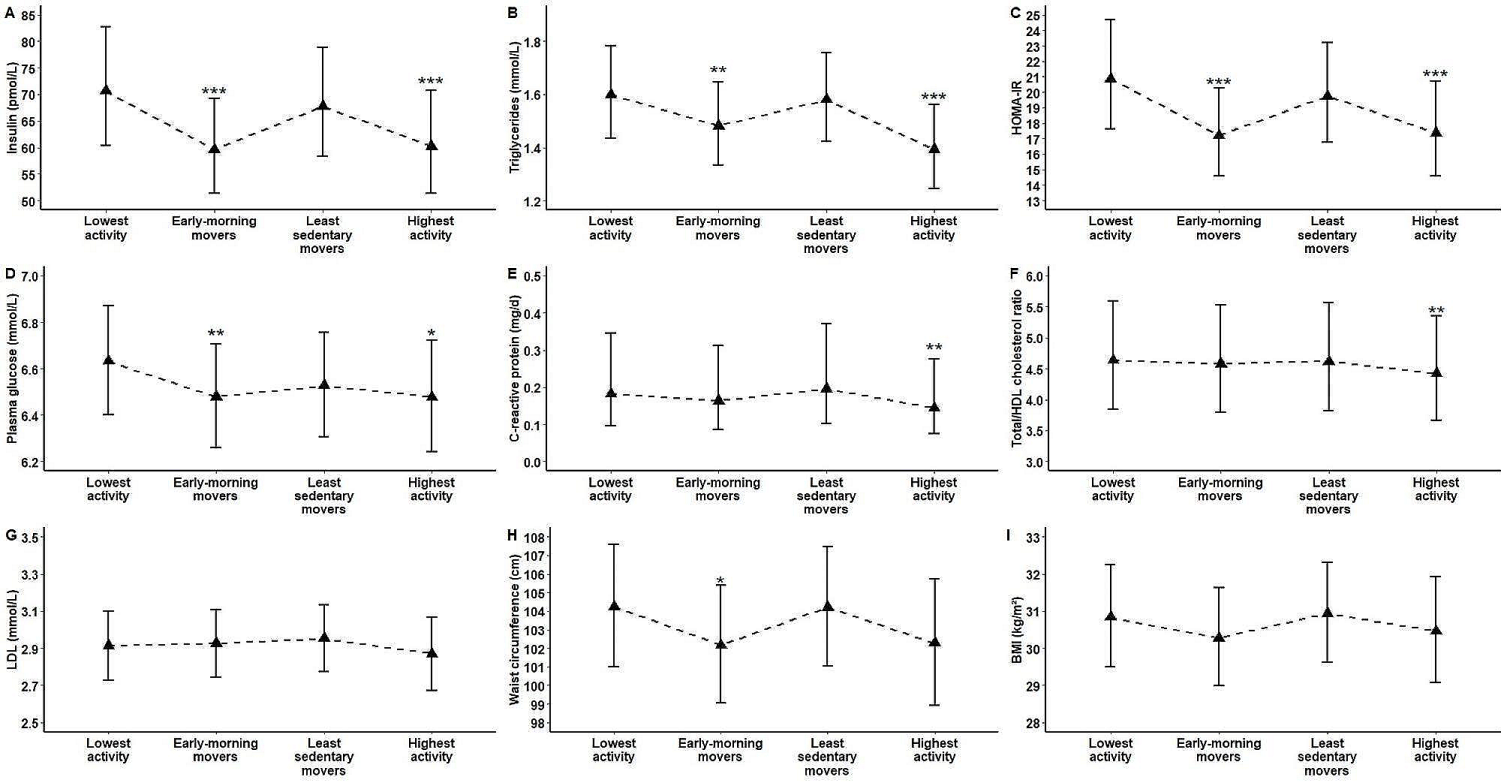
Association between the four identified profiles of movement behaviors with cardiometabolic biomarkers (A–I) with “Lowest activity” profile selected as the referent group. The values and error bars are estimated means and 95% confidence interval from linear regression models. All the markers except LDL (G) were back-transformed from the log scale. The models were adjusted for significant demographic, lifestyle, dietary, and medical history covariates identified through outcome-specific backward elimination (retained at *P* < 0.2: see Supplementary material, Table S1). ***P < 0.01; **P < 0.05; *P < 0.1. Abbreviations: BMI = body mass index, CRP = C-reactive protein, LDL = low-density lipoprotein, HDL = high-density lipoprotein, HOMA-IR = Homeostasis insulin resistance (HOMA-IR).

Figure 5 illustrates the results of the association analysis between the four distinct movement behavior profiles and markers of cardiometabolic health. After adjusting for potential covariates, “Early-morning movers” and the “Highest activity” profiles both had lower levels of insulin (*P* < 0.01 for both), triglycerides (*P* < 0.05 and *P* < 0.01, respectively), HOMA-IR (*P* < 0.01 for both), and plasma glucose (*P* < 0.05 and *P* < 0.1, respectively) compared to the “Lowest activity” profile. “Early-morning movers” profile had also lower waist circumference (*P* < 0.1), while the “Highest activity” profile had lower CRP (*P* < 0.05) and total/HDL cholesterol ratio (*P* < 0.05) compared to the “Lowest activity” profile.

The average differences between the “Early-morning movers” and “Highest activity” profiles and the “Lowest activity” profile were clinically meaningful [63] for insulin (11.0 and 10.4 pmol/L, respectively), triglycerides (0.12 and 0.2 mmol/L, respectively), and HOMA-IR (3.6 and 3.5, respectively). No significant differences in any of the examined cardiometabolic markers were observed for the “Least sedentary movers” profile compared to the “Lowest activity” profile.

## Discussion

In this study, the profiling of movement behaviors was performed using a novel approach employing a deep learning method applied on image representation of the entire accelerometer outputs recorded during wear time. Deep convolutional autoencoders were able to learn the image representation, encompassing the entire spectrum of movement behaviors. Cluster analysis based on the learned representations for the movement behavior images, resulted in identification of four distinct movement behavior profiles characterized by varying levels, timing, and patterns of accumulation of movement behaviors. These identified profiles were associated with the markers of cardiometabolic health.

To our knowledge, our study is the first to employ a deep learning approach to profile accelerometer-estimated movement behaviors, providing additional insights into the interplay between levels, timing and patterns of accumulation of movement behaviors and their relationship to cardiometabolic health. Our study has two main novelties. Firstly, we generated movement behavior images that encapsulate the entire waking movement behavior profile across seven measurement days from each participant into a single image. Secondly, we applied a deep clustering approach (i.e., convolutional autoencoder) on these images to obtain the movement behavior profiles. Compared to previous studies that have typically relied on predefined variables and thresholds for machine learning of movement behaviors [4, 11, 15, 17, 27], the deep learning-based profiles that were identified in our study are likely to more accurately represent natural patterns and timing of movement behaviors.

The deep learning-based profiles identified in our study not only exhibit diverse and varying durations of movement behaviors, but also had evident differences in patterns and temporality of accumulation of movement behaviors. These differences were found to be associated with the markers of cardiometabolic health, after adjustment for other potential confounders. These findings further strengthen the emerging evidence that underscores the multidimensional nature of movement behaviors [4, 9], highlighting the significance of considering the duration, timing, and patterns of accumulation of movement behaviors collectively, as they may all be related to the cardiometabolic health in adults.

It is currently well-documented that regular physical activity, even in small doses, is strongly associated with a reduction in cardiometabolic mortality and the risk of developing various chronic diseases [7]. However, large-scale population-based studies indicate that MVPA, on average, accounts for only 3–5% of adults’ movement behavior time throughout a 24-hour cycle, while sedentary and light-intensity activities constitute the major parts [64]. Recent research indicates all movement behaviors within waking hours may be interrelated, and codependently related to cardiometabolic health [21]. The identification of movement behavior profiles with differing duration of movement behaviors implicitly supports the findings of existing literature [21, 65], suggesting that all movement behaviors during the day are important and may be linked to cardiometabolic health.

Evident differences in the temporality of movement behaviors were observed among the identified movement behavior profiles. Currently, little is known about the underlying biological mechanisms by which timing of movement behaviors may influence health benefits, and even less about whether and how the timing and temporality light-intensity activities and sedentary behaviors could be related to different health indictors in adults. Still, epidemiological studies have continued to find that timing of movement behaviors, particularly exercise behaviors, may be related to cardiometabolic health and risk of mortality [27, 30, 32, 33]. However, most of previous studies have primarily focused on the timing of exercise, while neglecting the potential effects of timing other movement behaviors.

Deep learning of movement behavior profiles reveals that low sedentary time combined with a higher level of physical activity may not necessarily result in a better cardiometabolic health, which is the most advocated approach for improving health in adults [10]. Overall, the “Least sedentary movers” exhibited more favorable patterns and durations of movement behaviors than the “Early-morning movers”. However, when comparing them to the “Lowest activity” profile, “Least sedentary movers” with a midday pattern of physical activity did not show any significant differences in the examined cardiometabolic health markers. On the other hand, “Early-morning movers” who were engaged in the highest daily physical activity during the early morning had lower insulin level, triglycerides, HOMA-IR, plasma glucose, and waist circumference than the “Lowest activity” profile. The existing literature on the optimal timing for physical activity presents somewhat mixed results [66], although most studies utilizing device-based methods support the notion that morning physical activity may provide greater health benefits than afternoon, evening, or midday activity [30, 33]. For instance, a recent study involving over 85,000 individuals in the UK Biobank found that morning physical activity was linked to lower risks of incident cardiovascular diseases compared to participants with a midday pattern of physical activity [30]. These findings contribute to the current knowledge in the literature by emphasizing that, in addition to considering the volume and patterns of movement behaviors [30, 67], the timing of movement behaviors may also play a role in achieving maximal cardiometabolic health benefits.

Like “Early-morning movers” profile, the “Highest activity” profile had also lower levels of insulin, triglycerides, and plasma glucose compared to the “Lowest activity” profile. Our findings align with existing literature, which consistently shows that a higher level of physical activity is associated greater health benefits, even after accounting for the duration and patterns of accumulation of sedentary behaviors [12, 68]. Currently, it is still unclear whether optimal timing of movement behaviors combined with a relatively higher physical activity level can confer additional health benefits [32]. Further research is needed to fully understand the potential synergistic effects of timing and activity level on health outcomes. Nonetheless, our findings suggest that both a relatively higher level of physical activity and engaging in morning physical activity may potentially confer comparable cardiometabolic health benefits.

Our study has several notable strengths. Although advantageous, the proliferation of accelerometers has also been associated with several considerable challenges regarding how to address the complex underlying interdependencies between physical activity and sedentary behaviors to fully understand their health implications. A significant limitation of previous research on movement behaviors is that conventional measurement methods have not been sufficiently enriched to investigate all aspects of movement behaviors, including duration, patterns, and timing. Traditionally, only few averaged and aggregated values have been extracted from accelerometer signals [4, 9, 11, 17, 27]. The most common summary statistics calculated from accelerometer signals include mean time spent in different intensity categories, although other variables such as the number of sedentary interruptions have been also computed and studied [12, 28]. Nonetheless, such variables do not naturally depict the temporality and patterns of accumulation of movement behaviors, which have been demonstrated to be associated with health outcomes [27, 30, 32]. In our study, we employed a novel image representation of original accelerometer outputs to visually display the entire recorded movement behaviors, and used an innovative deep learning approach for profiling movement behaviors. Most importantly, image representation has transformed our problem from a signal processing challenge into a machine vision problem, allowing us to use advanced deep learning techniques. These techniques have consistently demonstrated excellent performance when applied to image data [36, 41]. Another notable benefit of representing accelerometer data as images was the ability to address the problem of non-wear periods. This issue can be particularly challenging when dealing with wearable data using machine learning approaches. By visually representing non-wear periods as areas with no color (represented as black), we were able to create images of the same size for everyone and deal with missing values. Our approach can be expanded to also incorporate participant characteristics and other potentially relevant data alongside latent variables for clustering analysis. Future studies may examine whether the addition of other types of data and participant characteristics could lead to better identification of movement behavior profiles.

Our study is not without limitations. The study design is observational and cross-sectional, which restricts the ability to establish causality for the observed associations. Therefore, further verification of our findings is warranted through prospective study designs. While similar profiles, such as low activity and movers [9, 12, 28], have been previously theorized and identified, additional studies with different populations are necessary to determine if similar profiles can be observed and if they are similarly associated with cardiometabolic health markers. It is important to note that the accelerometer used in this study was worn only during waking hours, and we did not consider the potential impact of sleep behaviors on cardiometabolic health. We employed a previously established method to distinguish between wear time and non-wear time [45]. To date, a number of algorithms have been proposed and validated for detecting non-wear time [69]. To the best of our knowledge, there is no universally accepted standard method for detecting non-wear time from accelerometer data. It is likely that employing a different algorithm for non-wear time detection could possibly generate different clusters from those identified. In recent years, the wearable accelerometers have also advanced significantly, providing the possibility of collecting high-resolution raw accelerometry signals around the clock [64], and subsequently a better data source for movement behavior research [64]. However, the NHANES accelerometry data utilized in the present study (2003/04 and 2005/06) were uniaxial activity counts, which can be considered as a limitation. In addition, although our data was limited to waking hours, our approach remains applicable to studies that gather 24-hour raw accelerometry data, a method increasingly used in recent research [64]. Activity images could potentially be generated from segmented raw acceleration data. This highlights the need for future studies to explore and apply deep learning methods in studies utilizing 24-hour raw accelerometer data. Given that sleep behavior may also be related to cardiometabolic health and waking movement behaviors, future studies utilizing 24-hour accelerometry data are needed to gain a deeper understanding of the role of sleep behavior as a component of the entire 24-hour day. Although deep learning structures have demonstrated the capability to learn data representations [41] even from incomplete images [70], it is likely that the incomplete parts in movement behavior images (i.e., shown in black) had a negative impact on the ability of convolutional autoencoders to accurately capture the image representations. While employing deep learning approaches have certain benefits, the inherent “black box” nature of these methods [41, 71] does not allow to realize the significance of duration, patterns, and timing in forming the movement behavior profiles. Exploring the relative importance of these aspects, possibly with explainable deep learning methods [71], requires further investigation in future studies.

In conclusion, our study utilized a novel deep learning approach to analyze movement behavior profiles and found four profiles that are representative of the natural patterns and timing of movement behaviors in everyday life. Our results highlight that the duration, timing, and patterns of accumulation of movement behaviors altogether may be related to cardiometabolic health markers in adults. Most importantly, deep learning of movement behaviors revealed that in addition to considering the duration and patterns of movement behaviors, the timing of physical activity may also be crucial for gaining additional health benefits.

### Electronic supplementary material

Below is the link to the electronic supplementary material.

Supplementary Material 1 - Learning curve of the convolutional autoencoder and the results of silhouette analysis

## Data Availability

NHANES data are openly available.

## References

[1] Nahavandi D, Alizadehsani R, Khosravi A, Acharya UR (2022). Application of artificial intelligence in wearable devices: opportunities and challenges. Comput Methods Programs Biomed.

[2] Wang X, Yu H, Kold S, Rahbek O, Bai S. Wearable sensors for activity monitoring and motion control: A review. Biomim Intell Robot [Internet]. 2023;3:100089. Available from: https://www.sciencedirect.com/science/article/pii/S2667379723000037.

[3] Mukhopadhyay SC (2014). Wearable sensors for human activity monitoring: a review. IEEE Sens J.

[4] Zheng X, Reneman MF, Preuper RHRS, Otten E, Lamoth CJC (2023). Relationship between physical activity and central sensitization in chronic low back pain: insights from machine learning. Comput Methods Programs Biomed.

[5] Sun S, Folarin AA, Zhang Y, Cummins N, Liu S, Stewart C et al. The utility of wearable devices in assessing ambulatory impairments of people with multiple sclerosis in free-living conditions. Comput Methods Programs Biomed [Internet]. 2022;227:107204. Available from: https://www.sciencedirect.com/science/article/pii/S0169260722005855. 10.1016/j.cmpb.2022.107204 36371974

[6] Sigcha L, Borzì L, Amato F, Rechichi I, Ramos-Romero C, Cárdenas A et al. Deep learning and wearable sensors for the diagnosis and monitoring of Parkinson’s disease: A systematic review. Expert Syst Appl [Internet]. 2023;229:120541. Available from: https://www.sciencedirect.com/science/article/pii/S0957417423010436.

[7] Khurshid S, Weng L-C, Nauffal V, Pirruccello JP, Venn RA, Al-Alusi MA (2022). Wearable accelerometer-derived physical activity and incident disease. NPJ Digit Med.

[8] Matias CN, Cardoso J, Cavaca ML, Cardoso S, Giro R, Vaz J et al. Game on: A cross-sectional study on gamers’ mental health, Game patterns, physical activity, eating and sleeping habits. Comput Human Behav [Internet]. 2023;148:107901. Available from: https://www.sciencedirect.com/science/article/pii/S0747563223002522.

[9] Farrahi V, Rostami M, Dumuid D, Chastin SFM, Niemelä M, Korpelainen R (2022). Joint profiles of sedentary time and physical activity in adults and their associations with Cardiometabolic Health. Med Sci Sport Exerc.

[10] Bull FC, Al-Ansari SS, Biddle S, Borodulin K, Buman MP, Cardon G (2020). World Health Organization 2020 guidelines on physical activity and sedentary behaviour. Br J Sports Med.

[11] Shim J, Fleisch E, Barata F. Wearable-based accelerometer activity profile as digital biomarker of inflammation, biological age, and mortality using hierarchical clustering analysis in NHANES 2011–2014. Sci Rep [Internet]. 2023;13:9326. 10.1038/s41598-023-36062-y. 10.1038/s41598-023-36062-y PMC10250365 37291134

[12] Farrahi V, Kangas M, Kiviniemi A, Puukka K, Korpelainen R, Jämsä T (2021). Accumulation patterns of sedentary time and breaks and their association with cardiometabolic health markers in adults. Scand J Med Sci Sports.

[13] Stamatakis E, Ahmadi MN, Gill JMR, Thøgersen-Ntoumani C, Gibala MJ, Doherty A et al. Association of wearable device-measured vigorous intermittent lifestyle physical activity with mortality. Nat Med. 2022;1–9. 10.1038/s41591-022-02100-x PMC9800274 36482104

[14] Chan A, Chan D, Lee H, Ng CC, Yeo AHL. Reporting adherence, validity and physical activity measures of wearable activity trackers in medical research: A systematic review. Int J Med Inform [Internet]. 2022;160:104696. Available from: https://www.sciencedirect.com/science/article/pii/S1386505622000107. 10.1016/j.ijmedinf.2022.104696 35121356

[15] Díaz C, Galy O, Caillaud C, Yacef K (2020). A Clustering Approach for modeling and analyzing changes in physical activity behaviors from accelerometers. IEEE Access.

[16] Nahavandi D, Alizadehsani R, Khosravi A. Integration of Machine Learning with Wearable technologies. Handb Human-Machine Syst. 2023;383–96.

[17] Chen M, Landré B, Marques-Vidal P, van Hees VT, van Gennip ACE, Bloomberg M (2023). Identification of physical activity and sedentary behaviour dimensions that predict mortality risk in older adults: development of a machine learning model in the Whitehall II accelerometer sub-study and external validation in the CoLaus study. eClinicalMedicine.

[18] Farrahi V, Rostami M. Machine learning in physical activity, sedentary, and sleep behavior research. J Act Sedentary Sleep Behav [Internet]. 2024;3:5. 10.1186/s44167-024-00045-9. 10.1186/s44167-024-00045-9 PMC11960357 40217437

[19] Niemelä M, Kiviniemi A, Ikäheimo TM, Tulppo M, Korpelainen R, Jämsä T et al. Compositional association of 24-hour movement behavior with incident major adverse cardiac events and all-cause mortality. Scand J Med Sci Sports [Internet]. 2023;33:641–50. Available from: https://onlinelibrary.wiley.com/doi/full/10.1111/sms.14315. 10.1111/sms.14315 36630572

[20] Farrahi V, Rostami M, Nauha L, Korpisaari M, Niemelä M, Jämsä T et al. Replacing sedentary time with physical activity and sleep: Associations with cardiometabolic health markers in adults. Scand J Med Sci Sports [Internet]. 2023;33:907–20. Available from: https://onlinelibrary.wiley.com/doi/10.1111/sms.14323. 10.1111/sms.14323 36703280

[21] Farrahi V, Kangas M, Walmsley R, Niemelä M, Kiviniemi A, Puukka K et al. Compositional associations of sleep and activities within the 24-h cycle with cardiometabolic health markers in adults. Med Sci Sports Exerc [Internet]. 2021;53:324–32. 10.1249/mss.0000000000002481. 10.1249/MSS.0000000000002481 PMC7879600 32776775

[22] Migueles JH, Aadland E, Andersen LB, Brønd JC, Chastin SF, Hansen BH (2022). GRANADA consensus on analytical approaches to assess associations with accelerometer-determined physical behaviours (physical activity, sedentary behaviour and sleep) in epidemiological studies. Br J Sports Med.

[23] Dumuid D, Pedišić Ž, Stanford TE, Martín-Fernández J-A, Hron K, Maher CA (2019). The compositional isotemporal substitution model: a method for estimating changes in a health outcome for reallocation of time between sleep, physical activity and sedentary behaviour. Stat Methods Med Res.

[24] Chastin SFM, Palarea-Albaladejo J, Dontje ML, Skelton DA (2015). Combined effects of time spent in physical activity, sedentary behaviors and sleep on obesity and cardio-metabolic health markers: a novel compositional data analysis approach. PLoS ONE.

[25] Gupta N, Hallman DM, Dumuid D, Vij A, Rasmussen CL, Jørgensen MB (2020). Movement behavior profiles and obesity: a latent profile analysis of 24-h time-use composition among Danish workers. Int J Obes.

[26] del Pozo Cruz B, McGregor DE, del Pozo Cruz J, Buman MP, Palarea-Albaladejo J, Alfonso-Rosa RM (2020). Integrating sleep, physical activity, and diet quality to estimate all-cause mortality risk: a combined compositional clustering and survival analysis of the NHANES 2005–2006 cycle. Am J Epidemiol.

[27] Niemelä M, Kangas M, Farrahi V, Kiviniemi A, Leinonen A-M, Ahola R (2019). Intensity and temporal patterns of physical activity and cardiovascular disease risk in midlife. Prev Med (Baltim).

[28] Verswijveren SJJ, Lamb KE, Leech RM, Salmon J, Timperio A, Telford RM (2020). Activity accumulation and cardiometabolic risk in youth: a latent profile approach. Med Sci Sport Exerc.

[29] Brady R, Brown WJ, Hillsdon M, Mielke GI. Patterns of accelerometer-measured physical activity and health outcomes in adults: a systematic review. Med Sci Sports Exerc. 2022. 10.1249/MSS.0000000000002900 35220369

[30] Albalak G, Stijntjes M, van Bodegom D, Jukema JW, Atsma DE, van Heemst D (2023). Setting your clock: associations between timing of objective physical activity and cardiovascular disease risk in the general population. Eur J Prev Cardiol.

[31] Aqeel M, Guo J, Lin L, Gelfand S, Delp E, Bhadra A et al. Temporal physical activity patterns are associated with obesity in U.S. adults. Prev Med (Baltim) [Internet]. 2021;148:106538. Available from: https://www.sciencedirect.com/science/article/pii/S0091743521001225. 10.1016/j.ypmed.2021.106538 PMC8489165 33798532

[32] Feng H, Yang L, Liang YY, Ai S, Liu Y, Liu Y (2023). Associations of timing of physical activity with all-cause and cause-specific mortality in a prospective cohort study. Nat Commun.

[33] Albalak G, Stijntjes M, Wijsman CA, Slagboom PE, van der Ouderaa FJ, Mooijaart SP (2022). Timing of objectively-collected physical activity in relation to body weight and metabolic health in sedentary older people: a cross-sectional and prospective analysis. Int J Obes.

[34] Wang J, Chen Y, Hao S, Peng X, Hu L (2019). Deep learning for sensor-based activity recognition: a survey. Pattern Recognit Lett.

[35] Chen X, Wang X, Zhang K, Fung K-M, Thai TC, Moore K et al. Recent advances and clinical applications of deep learning in medical image analysis. Med Image Anal [Internet]. 2022;79:102444. Available from: https://www.sciencedirect.com/science/article/pii/S1361841522000913. 10.1016/j.media.2022.102444 PMC9156578 35472844

[36] Liu X, Faes L, Kale AU, Wagner SK, Fu DJ, Bruynseels A (2019). A comparison of deep learning performance against health-care professionals in detecting diseases from medical imaging: a systematic review and meta-analysis. Lancet Digit Heal.

[37] Farrahi V, Niemelä M, Kärmeniemi M, Puhakka S, Kangas M, Korpelainen R (2020). Correlates of physical activity behavior in adults: a data mining approach. Int J Behav Nutr Phys Act.

[38] Farrahi V, Niemelä M, Tjurin P, Kangas M, Korpelainen R, Jämsä T (2020). Evaluating and enhancing the generalization performance of machine learning models for physical activity intensity prediction from raw acceleration data. IEEE J Biomed Heal Inf.

[39] Chong J, Tjurin P, Niemelä M, Jämsä T, Farrahi V (2021). Machine-learning models for activity class prediction: a comparative study of feature selection and classification algorithms. Gait Posture.

[40] Behrad F, Saniee Abadeh M. An overview of deep learning methods for multimodal medical data mining. Expert Syst Appl [Internet]. 2022;200:117006. Available from: https://www.sciencedirect.com/science/article/pii/S0957417422004249.

[41] Egger J, Gsaxner C, Pepe A, Pomykala KL, Jonske F, Kurz M et al. Medical deep learning—A systematic meta-review. Comput Methods Programs Biomed [Internet]. 2022;221:106874. Available from: https://www.sciencedirect.com/science/article/pii/S0169260722002565. 10.1016/j.cmpb.2022.106874 35588660

[42] Li P, Pei Y, Li J. A comprehensive survey on design and application of autoencoder in deep learning. Appl Soft Comput [Internet]. 2023;138:110176. Available from: https://www.sciencedirect.com/science/article/pii/S1568494623001941.

[43] Ige AO, Noor MHM. A survey on unsupervised learning for wearable sensor-based activity recognition. Appl Soft Comput. 2022;109363.

[44] National Health and Nutrition Examination Survey [Internet]. Centers Dis. Control Prev. (CDC), Natl. Cent. Heal. Stat. [cited 2023 May 23]. Available from: https://www.cdc.gov/nchs/nhanes/.

[45] Matthews CE, Chen KY, Freedson PS, Buchowski MS, Beech BM, Pate RR (2008). Amount of time spent in sedentary behaviors in the United States, 2003–2004. Am J Epidemiol.

[46] Leroux A, Di J, Smirnova E, Mcguffey EJ, Cao Q, Bayatmokhtari E (2019). Organizing and analyzing the activity data in NHANES. Stat Biosci.

[47] Freedson PS, Melanson E, Sirard J (1998). Calibration of the Computer Science and Applications, Inc. accelerometer. Med Sci Sports Exerc.

[48] Yerramalla MS, van Hees VT, Chen M, Fayosse A, Chastin SFM, Sabia S (2022). Objectively measured total sedentary time and pattern of sedentary accumulation in older adults: associations with incident cardiovascular disease and all-cause mortality. Journals Gerontol Ser A.

[49] Niemelä M, Kiviniemi A, Kangas M, Farrahi V, Leinonen A-M, Ahola R et al. Prolonged bouts of sedentary time and cardiac autonomic function in midlife. Transl Sport Med [Internet]. 2019;2:341–50. Available from: https://onlinelibrary.wiley.com/doi/10.1002/tsm2.100.

[50] Chastin SFM, Egerton T, Leask C, Stamatakis E (2015). Meta-analysis of the relationship between breaks in sedentary behavior and cardiometabolic health. Obesity.

[51] Colvin A, Murray L, Noble J, Chastin S. Effects of breaking up sedentary behavior with short bouts of yoga and Tai-Chi on Glycemia, Concentration, and well-being. J Phys Act Health. 2023;1–8. 10.1123/jpah.2023-0308 37992705

[52] Tremblay MS, Aubert S, Barnes JD, Saunders TJ, Carson V, Latimer-Cheung AE et al. Sedentary behavior research network (SBRN) - terminology consensus project process and outcome. Int J Behav Nutr Phys Act. 2017;14. 10.1186/s12966-017-0525-8 PMC5466781 28599680

[53] Lyden K, Kozey Keadle SL, Staudenmayer JW, Freedson PS (2012). Validity of two wearable monitors to estimate breaks from sedentary time. Med Sci Sports Exerc.

[54] Millán J, Pintó X, Muñoz A, Zúñiga M, Rubiés-Prat J, Pallardo LF (2009). Lipoprotein ratios: physiological significance and clinical usefulness in cardiovascular prevention. Vasc Health Risk Manag.

[55] Wallace TM, Levy JC, Matthews DR (2004). Use and abuse of HOMA modeling. Diabetes Care.

[56] Guo X, Liu X, Zhu E, Yin J. Deep clustering with convolutional autoencoders. Neural Inf Process 24th Int Conf ICONIP 2017, Guangzhou, China, Novemb 14–18, 2017, Proceedings, Part II 24. 2017. p. 373–82.

[57] Längkvist M, Karlsson L, Loutfi A (2014). A review of unsupervised feature learning and deep learning for time-series modeling. Pattern Recognit Lett.

[58] Kanungo T, Mount DM, Netanyahu NS, Piatko CD, Silverman R, Wu AY (2002). An efficient k-means clustering algorithm: analysis and implementation. IEEE Trans Pattern Anal Mach Intell.

[59] Arthur D, Vassilvitskii S. K-means + + the advantages of careful seeding. Proc eighteenth Annu ACM-SIAM Symp Discrgorithms. 2007. p. 1027–35.

[60] Kodinariya TM, Makwana PR (2013). Review on determining number of cluster in k-means clustering. Int J Adv Res Comput Sci Manag Stud.

[61] Shahapure KR, Nicholas C. Cluster quality analysis using silhouette score. 2020 IEEE 7th Int Conf Data Sci Adv Anal. 2020. p. 747–8.

[62] Healy GN, Matthews CE, Dunstan DW, Winkler EAH, Owen N (2011). Sedentary time and cardio-metabolic biomarkers in US adults: NHANES 2003-06. Eur Heart J.

[63] Group DIS (2004). Plasma insulin and cardiovascular mortality in non-diabetic European men and women: a meta-analysis of data from eleven prospective studies. Diabetologia.

[64] Rosenberger ME, Fulton JE, Buman MP, Troiano RP, Grandner MA, Buchner DM (2019). The 24-hour activity cycle: a new paradigm for physical activity. Med Sci Sports Exerc.

[65] Chastin SFM, McGregor DE, Biddle SJH, Cardon G, Chaput J-P, Dall PM (2021). Striking the right balance: evidence to inform combined physical activity and sedentary behavior recommendations. J Phys Act Heal.

[66] Janssen I, Campbell JE, Zahran S, Saunders TJ, Tomasone JR, Chaput J-P (2022). Timing of physical activity within the 24-hour day and its influence on health: a systematic review. Heal Promot Chronic Dis Prev Can Res Policy Pract.

[67] van der Velde JHPM, Boone SC, Winters-van Eekelen E, Hesselink MKC, Schrauwen-Hinderling VB, Schrauwen P (2023). Timing of physical activity in relation to liver fat content and insulin resistance. Diabetologia.

[68] Chastin SFM, De Craemer M, De Cocker K, Powell L, Van Cauwenberg J, Dall P (2019). How does light-intensity physical activity associate with adult cardiometabolic health and mortality? Systematic review with meta-analysis of experimental and observational studies. Br J Sports Med.

[69] Banda JA, Haydel KF, Davila T, Desai M, Bryson S, Haskell WL (2016). Effects of varying epoch lengths, wear time algorithms, and activity cut-points on estimates of child sedentary behavior and physical activity from accelerometer data. PLoS ONE.

[70] Zhao J, Lv Y, Zhou Z, Cao F (2017). A novel deep learning algorithm for incomplete face recognition: low-rank-recovery network. Neural Netw.

[71] Loh HW, Ooi CP, Seoni S, Barua PD, Molinari F, Acharya UR (2022). Application of explainable artificial intelligence for healthcare: a systematic review of the last decade (2011–2022). Comput Methods Programs Biomed.

