# Supplementary material for: Deep learning of movement behavior profiles and their association with markers of cardiometabolic health
Source: BMC Med Inform Decis Mak. 2024 Mar 13;24:74. doi: 10.1186/s12911-024-02474-7 (PMC10936042; doi:10.1186/s12911-024-02474-7)
Supplement: Supplementary file 1 — Supplementary Material 1 - Learning curve of the convolutional autoencoder and the results of silhouette analysis [file 12911_2024_2474_MOESM1_ESM.docx]

**
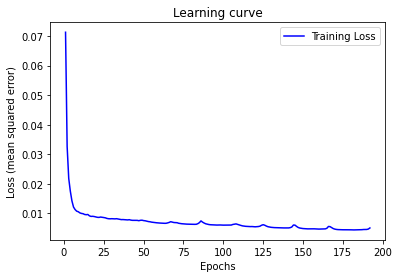
**

**Supplementary Figure 1**: Learning curve of the convolutional autoencoder with 32 latent variables, trained for 200 epochs, showing the mean squared error (MSE) on the dataset during containing all the movement behavior profile images (N = 1812).

**
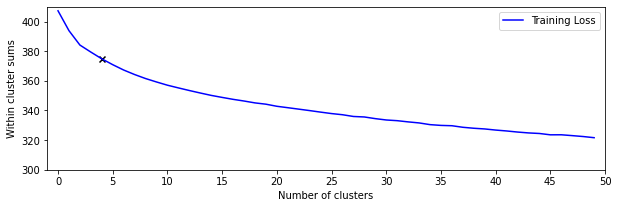
Supplementary Figure 2**: Within-cluster sums for K-means cluster analysis with number of clusters ranging from 1 to 50. + is the optimal number of clusters (K = 4) according the ‘elbow method’.

**Results of silhouette analysis.**

Silhouette analysis was conducted with the number of clusters ranging from 2 to 10. Results for clusters exceeding 10 were not presented, as they lacked meaningful distinctions.

**
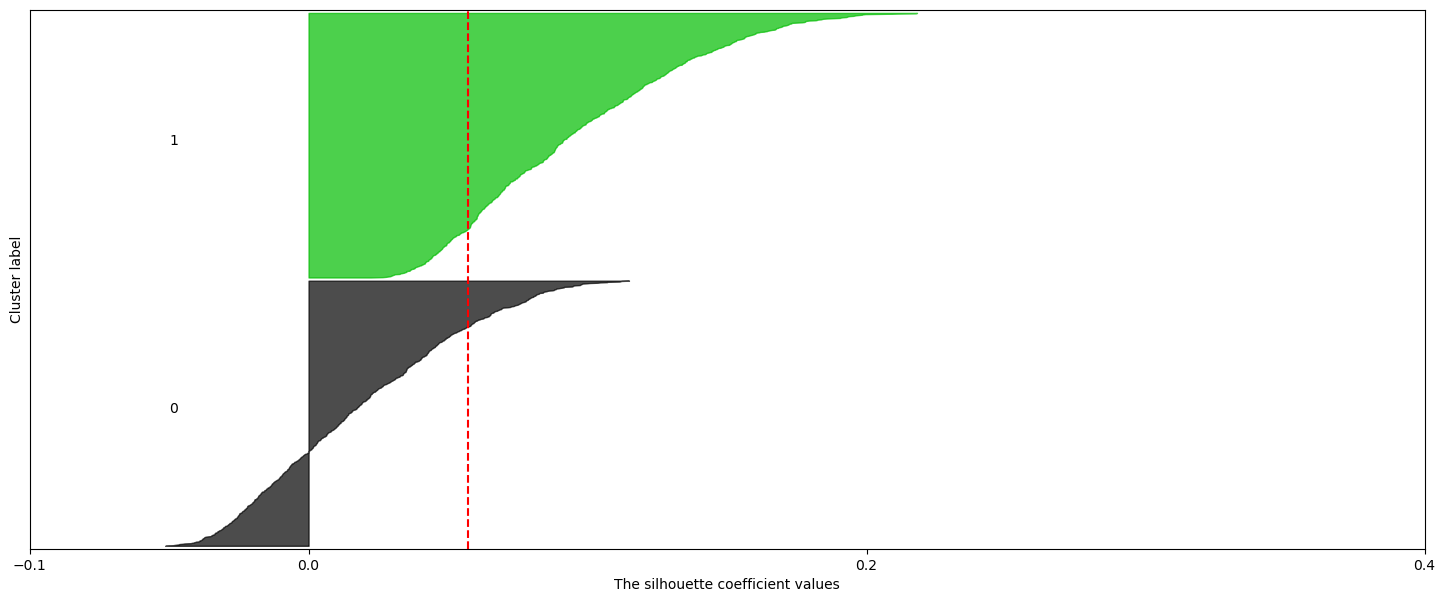
**

**Supplementary Figure 3**: The silhouette score was computed for each participant (activity image) in the dataset with the number of clusters set to K=2. The average silhouette score was found to be 0.057124343.


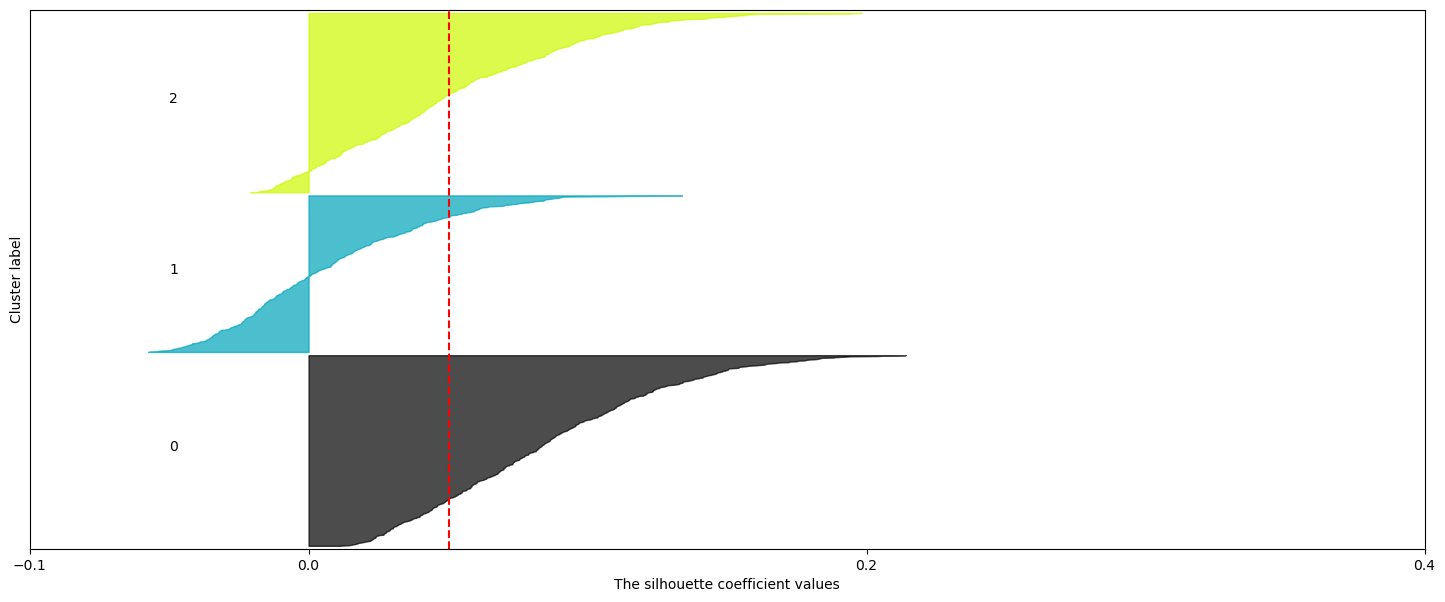


**Supplementary Figure 4**: The silhouette score was computed for each participant (activity image) in the dataset with the number of clusters set to K=3. The average silhouette score was found to be 0.0502837.

**
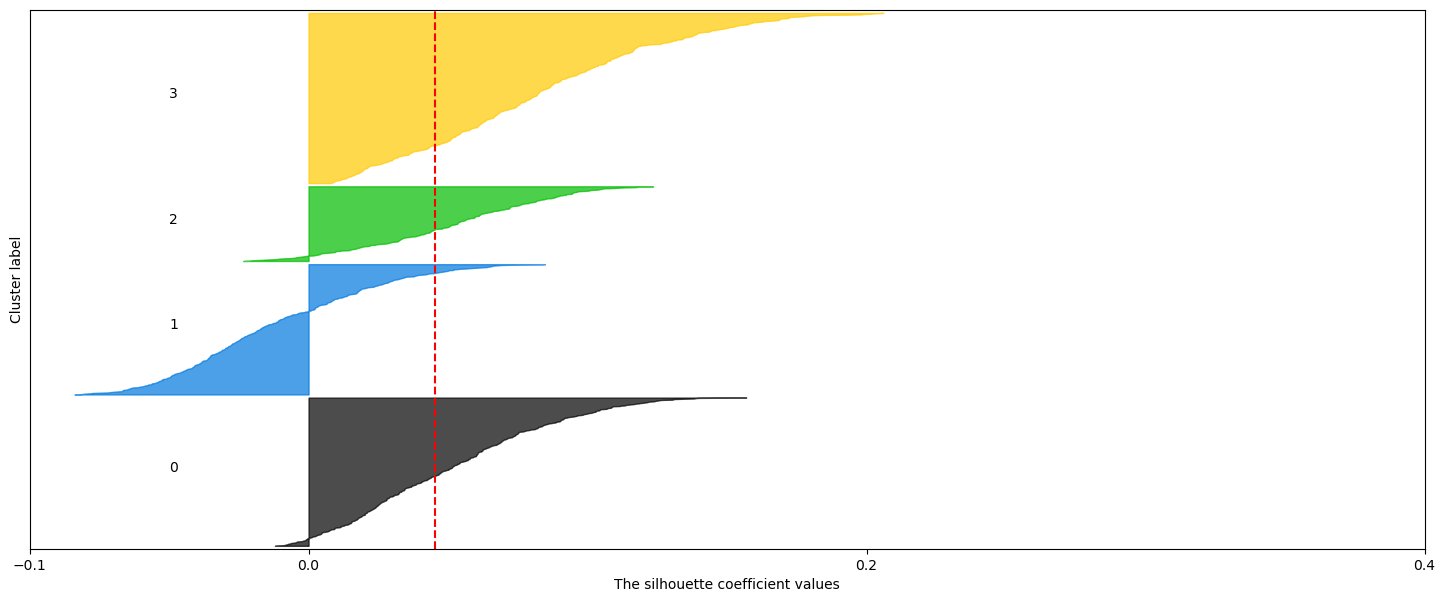
Supplementary Figure 5**: The silhouette score was computed for each participant (activity image) in the dataset with the number of clusters set to K=4. The average silhouette score was found to be 0.04543108.


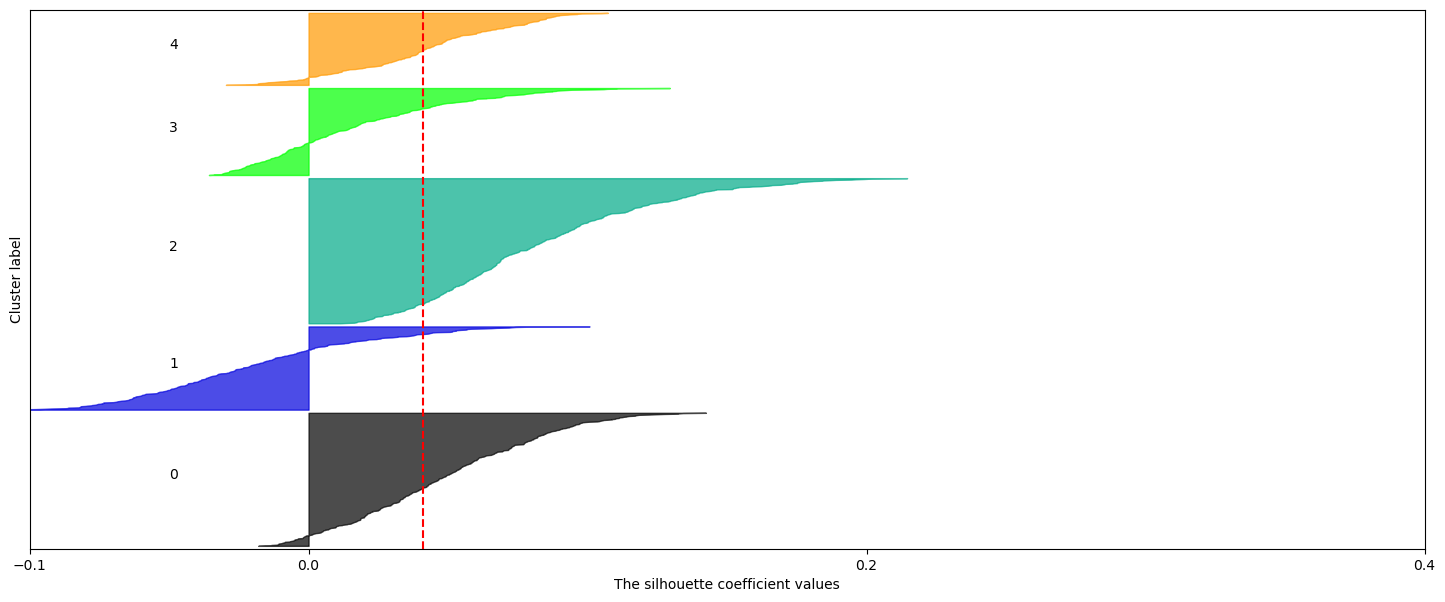


**Supplementary Figure 6**: The silhouette score was computed for each participant (activity image) in the dataset with the number of clusters set to K=5. The average silhouette score was found to be 0.04115946.


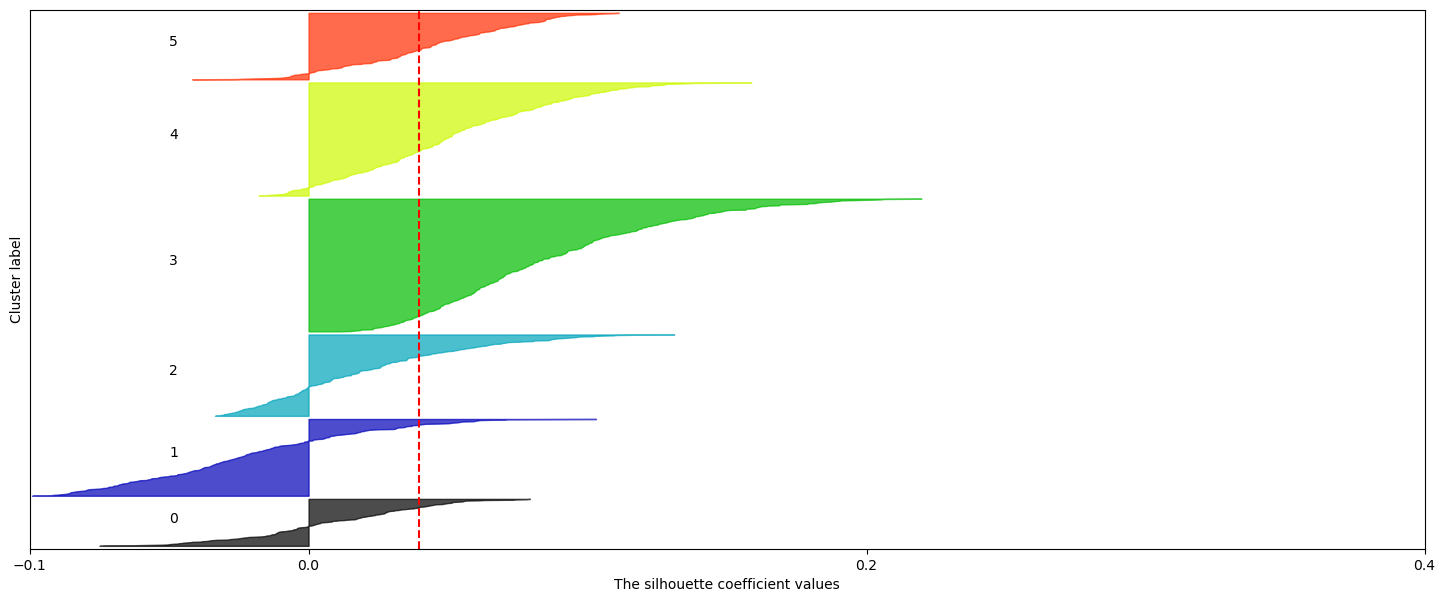


**Supplementary Figure 7**: The silhouette score was computed for each participant (activity image) in the dataset with the number of clusters set to K=6. The average silhouette score was found to be 0.039635483


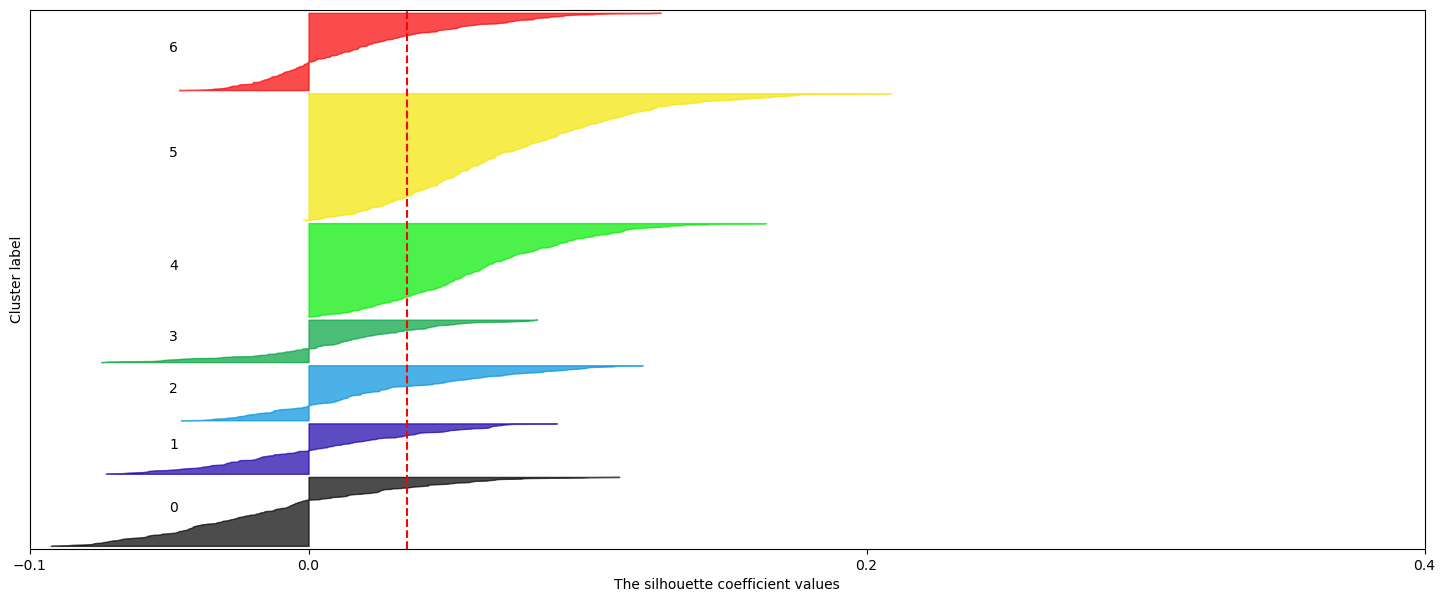


**Supplementary Figure 8**: The silhouette score was computed for each participant (activity image) in the dataset with the number of clusters set to K=7. The average silhouette score was found to be 0.03525374.

**
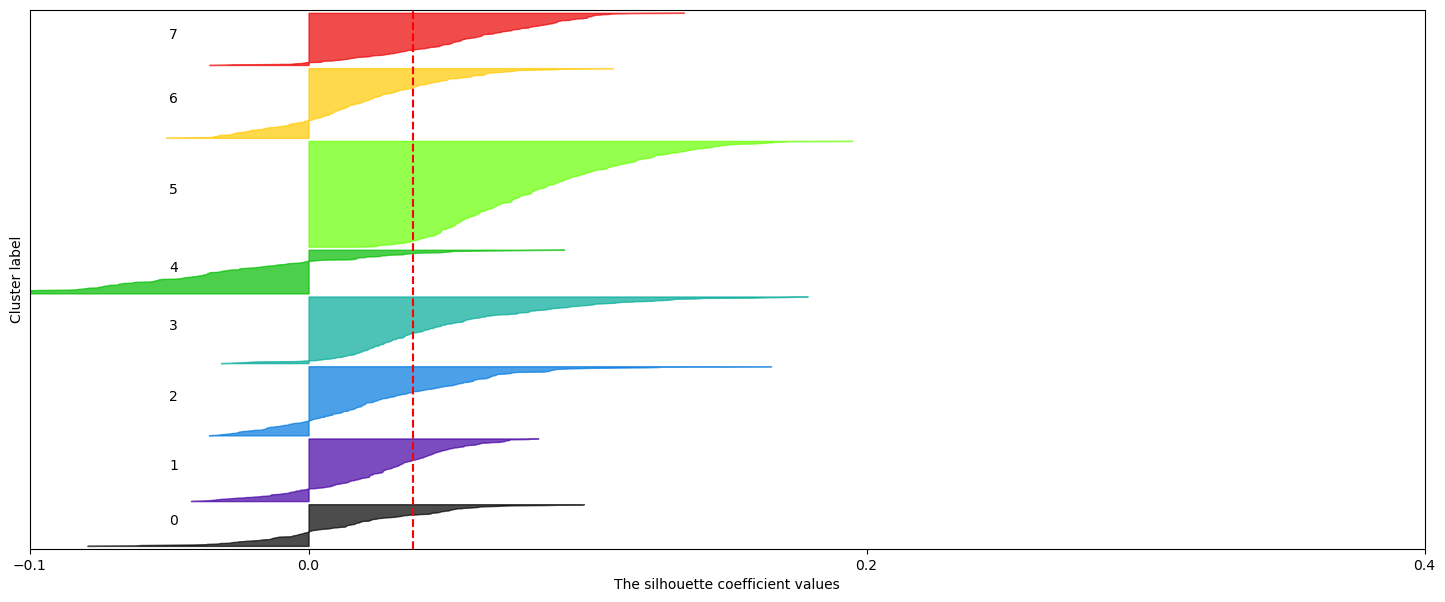
**

**Supplementary Figure 9**: The silhouette score was computed for each participant (activity image) in the dataset with the number of clusters set to K=8. The average silhouette score was found to be 0.037612896.


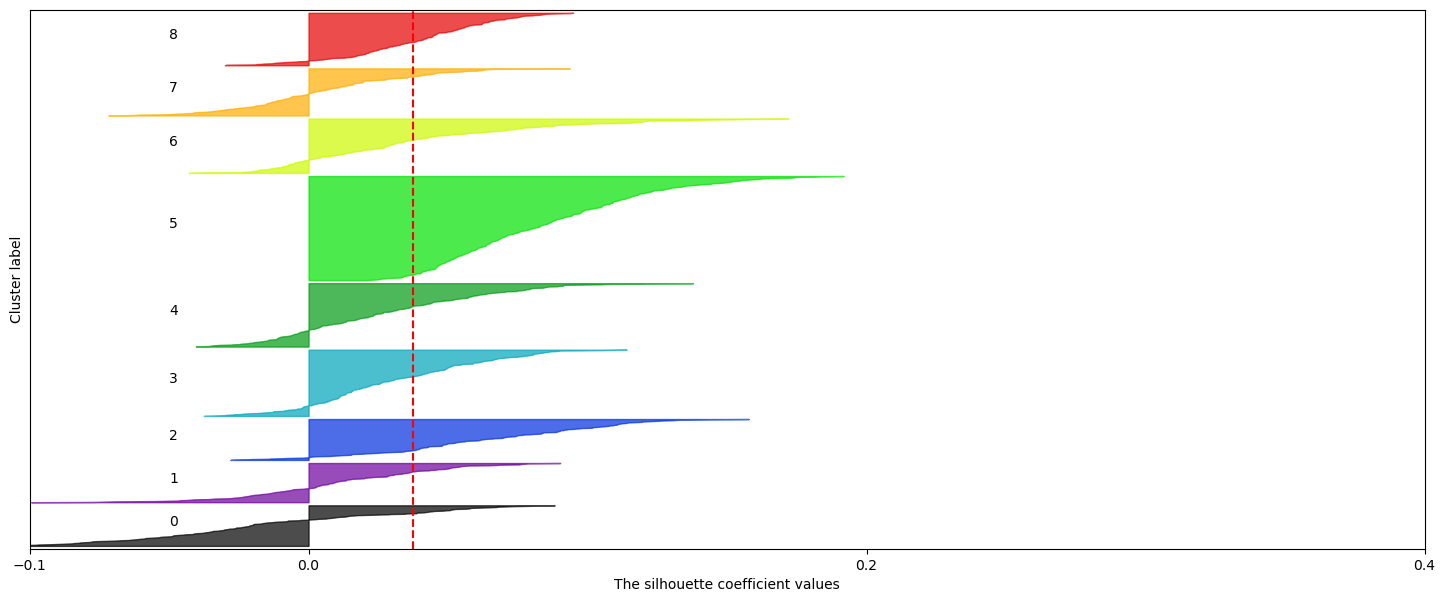


**Supplementary Figure 10**: The silhouette score was computed for each participant (activity image) in the dataset with the number of clusters set to K=9. The average silhouette score was found to be 0.037489552


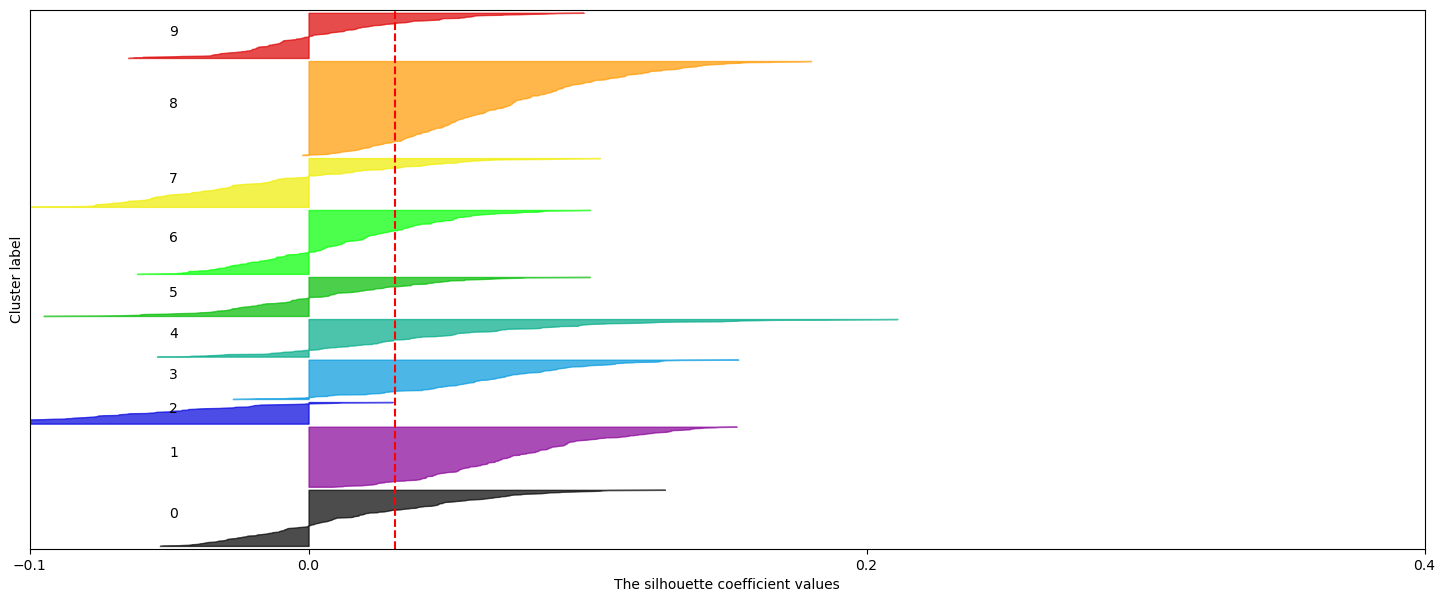


**Supplementary Figure 11**: The silhouette score was computed for each participant (activity image) in the dataset with the number of clusters set to K=10. The average silhouette score was found to be 0.031062221.

**Supplementary Table 1:** Covariates retained through backward elimination in association analysis with cardiometabolic markers.

| Cardiometabolic marker | Covariates retained through backward elimination |
| --- | --- |
| Insulin | Gender, ethnicity, education level, Income to poverty ratio, smoking, alcohol consumption, total saturated fat, total caffeine intake, self-reported cardiovascular diseases, self-reported diabetes |
| Triglycerides | Age, gender, ethnicity, marital status, education level, smoking, alcohol consumption, dietary energy intake, total saturated fat, total caffeine intake, self-reported cardiovascular diseases, self-reported diabetes |
| HOMA-IR | Age, gender, ethnicity, education level, income to poverty ration, smoking, alcohol consumption, total saturated fat, total caffeine intake, self-reported cardiovascular diseases, self-reported diabetes |
| Plasma glucose | Age, gender, ethnicity, marital status, education level, smoking, dietary energy intake, total saturated fat, self-reported cardiovascular diseases, self-reported diabetes |
| CRP | Age, gender, ethnicity, marital status, income to poverty ratio, alcohol consumption, total saturated fat, total caffeine intake, self-reported diabetes |
| Total/HDL cholesterol ratio | Age, gender, ethnicity, education level, smoking, alcohol consumption, dietary energy intake, total saturated fat, self-reported cardiovascular diseases, cancer |
| LDL | Age, gender, ethnicity, education level, smoking, alcohol consumption, self-reported cardiovascular diseases, self-reported diabetes |
| Waist circumference | Age, gender, ethnicity, marital status, education level, smoking, alcohol consumption, total saturated fat, self-reported cardiovascular diseases, self-reported diabetes |
| BMI | Age, gender, ethnicity, marital status, education level, smoking, alcohol consumption, dietary energy intake, total saturated fat, total caffeine intake, self-reported cardiovascular diseases, self-reported diabetes |

BMI = body mass index, CRP = C-reactive protein, LDL = low-density lipoprotein, HDL = high-density lipoprotein, HOMA-IR = Homeostasis insulin resistance (HOMA-IR).
